# Size Selectivity in Heterolanthanide Molecular Complexes with a Ditopic Ligand

**DOI:** 10.1002/chem.202202823

**Published:** 2022-11-24

**Authors:** Luca Bellucci, Lorenzo Fioravanti, Lidia Armelao, Gregorio Bottaro, Fabio Marchetti, Francesco Pineider, Giordano Poneti, Simona Samaritani, Luca Labella

**Affiliations:** ^1^ Dipartimento di Chimica e Chimica Industriale and CIRCC Università di Pisa via Giuseppe Moruzzi 13 56124 Pisa Italy; ^2^ Dipartimento di Scienze Chimiche Università di Padova via Marzolo 1 35131 Padova Italy; ^3^ CNR ICMATE and INSTM Dipartimento di Scienze Chimiche Università di Padova via Marzolo 1 35131 Padova Italy; ^4^ Dipartimento di Scienze Chimiche e Tecnologie dei Materiali (DSCTM) Consiglio Nazionale delle Ricerche Piazzale A. Moro 7 00185 Roma Italy; ^5^ Instituto de Química Universidade Federal do Rio de Janeiro Avenida Athos da Silveira Ramos, 149 Centro de Tecnologia – Cidade Universitária 21941-909 Rio de Janeiro Brazil

**Keywords:** hetero-lanthanides complexes, lanthanides, luminescence, magnetometry, X-ray diffraction

## Abstract

The similar reactivity of lanthanides generally leads to statistically populated polynuclear complexes, making the rational design of ordered hetero‐lanthanide compounds extremely challenging. Here we report on the site selectivity in hetero‐lanthanide tetranuclear complexes afforded by the relatively simple ditopic pyterpyNO ligand (4’‐(4‐pyridil)‐2,2’:6’,2”‐terpyridine *N*‐oxide). The sequential room temperature reaction of RE_2_(tta)_6_(pyterpyNO)_2_ (where RE=Y, (**1)**; Eu, (**2**), Dy, (**3**) Htta=2‐thenoyltrifluoroacetone) with La(tta)_3_dme (dme=dimethoxyethane) yielded Y_2_La_2_(tta)_12_(pyterpyNO)_2_ (**4**), Dy_2_La_2_(tta)_12_(pyterpyNO)_2_ (**5**) and Eu_2_La_2_(tta)_12_(pyterpyNO)_2_ (**6**). Single crystals X‐ray diffraction studies showed that **4, 5** and **6** are isostructural, featuring a tetranuclear structure with two different metal coordination sites with coordination numbers 8 (CN8) and 9 (CN9). The two smaller cations are mainly bridged by the *O*‐donor atoms of the NO groups of two pyterpyNO ligands (CN8), while the larger lanthanum centres are bound by a terpyridine unit (CN9). Size selectivity has been studied with structural and magnetic studies in the solid state and through ^19^F NMR and photoluminescence studies in solution, showing a direct dependence on the difference of ionic radii of the ions and yielding a 91 % selectivity for **4**. Furthermore, ^19^F NMR, X‐ray and PL studies pointed out that the nature of the product is independent from the synthetic route for compound Eu_2_Y_2_(tta)_12_(pyterpyNO)_2_ (**7**), keeping the ion selectivity also for a self‐assembly reaction. Unexpectedly, these studies have evidenced that selectivity is not exclusively governed by electrostatic interactions related to size dimensions.

## Graphical abstract

**A size selectivity in** the coordination of different lanthanides ions to the two donor sites of the ditopic pyterpyNO ligand has been evidenced in heteronuclear complexes of formula RE_2_RE*_2_(tta)_12_(pyterpyNO)_2_, where smaller (larger) ions normally display a higher affinity for the eight (nine)‐coordinated oxygen (nitrogen) site. The present results, obtained in solution and in the solid state with a multi‐technique approach (X‐ray diffraction, ^19^F NMR, magnetometry and photoluminescence), are independent of the employed synthetic route.

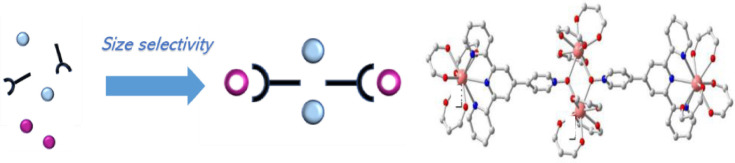

## 1 Introduction

The electronic configuration of lanthanide ions, featuring a 4f incomplete shell shielded from the interaction with the surrounding chemical species, makes them appealing for the development of functional materials with several fields of applicability (luminescence, magnetism, catalysis, among the others).[^1^, ^2^] On this regard, the coexistence of different lanthanide ions in the same context has been frequently sought to prepare multifunctional materials^[3]^ with tuneable properties in luminescence,[^4^, ^5^, ^6^, ^7^, ^8^, ^9^, ^10^] magnetism[^11^, ^12^, ^13^, ^14^, ^15^] and catalysis,[^16^, ^17^, ^18^] featuring potential applications for dual emissions and up‐conversion,[^19^, ^20^, ^21^, ^22^, ^23^, ^24^] multiple signal detection,^[25]^ barcoded materials,[^26^, ^27^] bioimaging nanoprobes,[^28^, ^29^] and as units of information for quantum computation.^[30]^ However, the similarity in chemical reactivity across the series and their high kinetic lability make the separation of lanthanide ions, as well as the preparation of ordered hetero‐lanthanide systems, challenging. As such, while hetero‐lanthanide complexes with two or more statistically distributed lanthanide centres are largely present in the literature, ordered heterometallic complexes, featuring different metals in different sites, are far less common, and only very few hetero‐lanthanide molecular architectures having enough stability to avoid scrambling have been reported so far.[^31^, ^32^] Polynuclear architectures with inequivalent and unscrambled coordination sites[^33^, ^34^, ^35^, ^36^, ^37^, ^38^, ^39^, ^40^, ^41^] have been prepared employing structurally complicated flexible multidentate ligands or multi‐compartmental macrocycles, often requiring a long and elaborate synthesis, relying on the size selectivity offered by the effects of the well‐known lanthanide contraction.[^42^, ^43^, ^44^] In one case, a simpler ligand (8‐hydroxyquinoline, Q) has been shown to self‐assemble heterometallic [NdLn_2_(Q)_9_] (Ln=Er, Yb) species, where the larger neodymium is well discriminated from the two other smaller ions, reaching a 90 % size selectivity.^[45]^ A different approach, involving preformed building blocks of kinetically inert lanthanide macrocyclic complexes, has been adopted to obtain ordered hetero‐lanthanide structures.[^46^, ^47^, ^48^, ^49^] In this case, the synthetic strategy relies on a two‐step sequence of reactions, each one involving a single lanthanide centre,[^50^, ^51^, ^52^, ^53^, ^54^, ^55^, ^56^] where sufficiently inert monometallic complexes have to be reacted selectively with the second lanthanide ion. Triple‐decker porphyrin and phthalocyanine complexes are obtained in this way.[^57^, ^58^, ^59^, ^60^, ^61^, ^62^, ^63^, ^64^] Moreover, using this stepwise synthetic approach, an ordered sequence of europium and terbium centres has been grafted onto a surface and characterized through PL studies.^[65]^

**Scheme 1:**
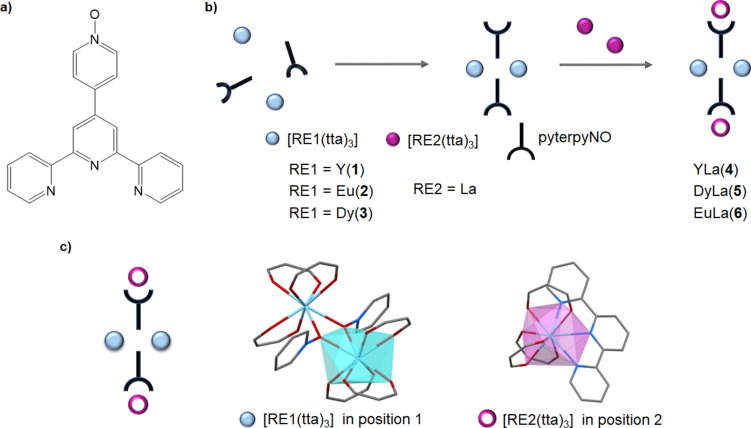
**a)** 4’‐(4’’’‐pyridyl‐N‐oxide)‐2 : 2’,6’:2’’‐terpyridine (pyterpyNO), **b)** Sequential synthetic approach for the synthesis of heterolanthanide tetranuclear complexes **c)** Coordination polyhedra. Light blue: Distorted dodecahedron of *O*‐coordinated REl. Magenta: Tricapped trigonal prism of *N*‐coordinated RE2.

In this work, we propose a ditopic ligand, 4’‐(4’’’‐pyridyl‐*N*‐oxide)‐2 : 2’,6’:2’’‐terpyridine, (pyterpyNO; Scheme 1a), featuring two coordination sites with different structural and chemical properties, to impose a selectivity on the coordination of different lanthanide ions. This rigid aromatic ligand (Scheme 1a) possesses two binding sites: the NO group, as in the analogous pyrazine *N*‐oxide or 4,4’bipyridine *N*‐oxide (bipyMO) ligands,[^66^, ^67^, ^68^, ^69^] and the terpyridine moiety. The former site has shown a marked preference for *O*‐coordination to lanthanides so that a careful control of the metal/ligand stoichiometry afforded dinuclear RE_2_(tta)_6_(pyterpyNO)_2_ complexes with an uncoordinated terpyridine (Scheme 1b). The terpyridine bonding site, however, can be employed for the coordination of a different RE*(tta)_3_ fragment, so that the dinuclear precursor is potentially useful to produce hetero‐lanthanide molecular complexes.^[70]^ The two binding sites present different coordination numbers, leading to polyhedra with significantly different volumes, which can be employed to exert size selectivity in the coordination of lanthanide ions. In the homo‐metallic dysprosium compound, where differences are due only to the coordination geometry, the octa‐coordinated site has a volume of 23.26(4) Å^3^ while the nona‐coordinated site, a volume of 29.19(5) Å^3^.^[71]^ As a consequence, smaller ions are expected to prefer the oxygen coordination (CN8; position 1) while larger ions the terpyridine moiety (CN9; position 2). In this work we demonstrate, through a multi‐technique approach, that the pyterpyNO ligand is able to coordinate different lanthanide ions with a selectivity independent of the employed synthetic route, in the solution phase as well in the crystalline phase, peaking 91 % selectivity for Y_2_La_2_(tta)_12_(pyterpyNO)_2_ (**4**).

## 2 Results

### 2.1 Synthesis, crystallography and NMR studies

**Figure 1:**
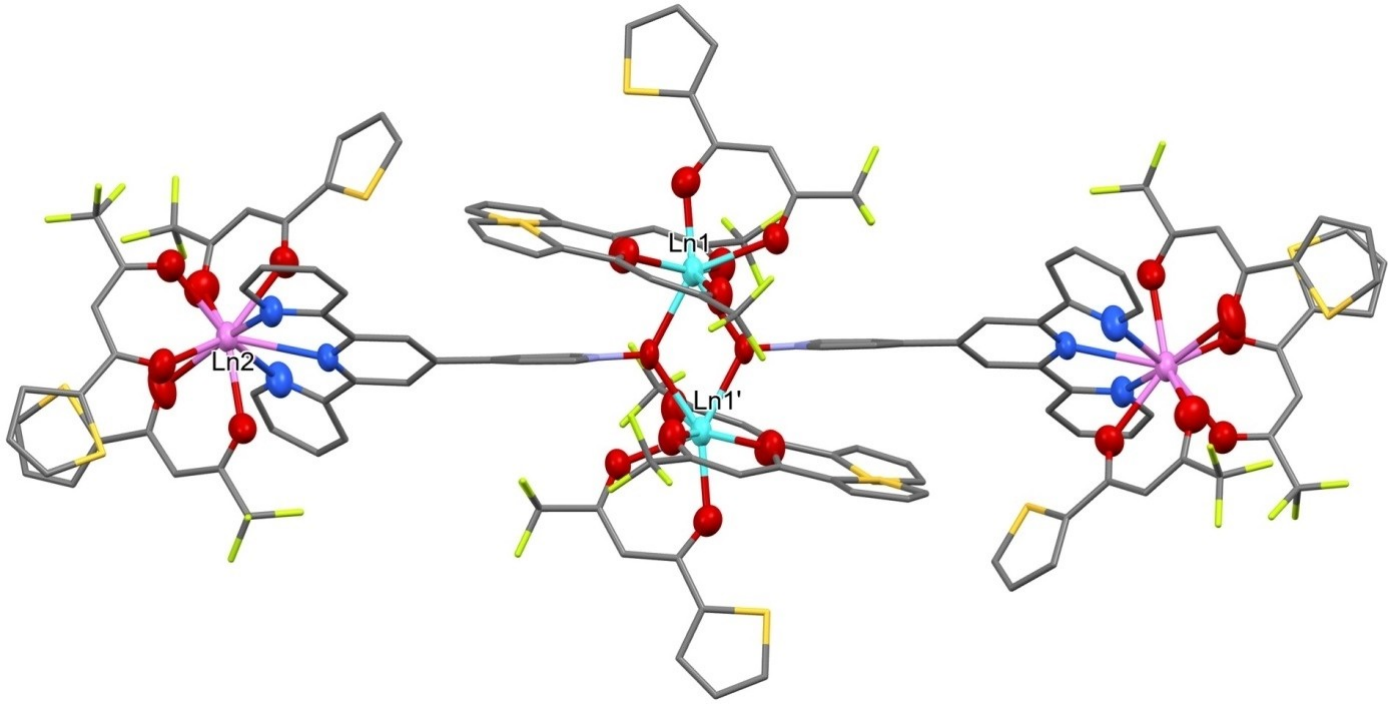
The centrosymmetric structure of the tetranuclear compounds described in this paper. The two different positions of the lanthanide metals are labelled.

A toluene solution of **1** quickly and cleanly reacts at room temperature with two equivalents of the diamagnetic La(tta)_3_(dme), as monitored via NMR spectroscopy from the appearance of the free dme signals in the resulting solution as already reported in the synthesis of Y_4_(tta)_12_(pyterpyNO)_2_.^[70]^ IR spectroscopy supports the formation of the tetranuclear complex Y_2_La_2_(tta)_12_(pyterpyNO)_2_ (**4**), since its spectrum is almost completely superimposable with the one from the yttrium tetranuclear compound (Figure S1). Elemental analysis is consistent with a molar ratio of the two metals of 1. Slow evaporation of a toluene solution yielded well‐shaped single crystals,^[72]^ suitable for X‐ray analysis (Figure 1), isotypic with the homometallic tetranuclear dysprosium analogue Dy_4_(tta)_12_(pyterpyNO)_2_.^[70]^ The expected size selectivity should produce here a preference for yttrium in the internal position (position 1 in Figure 1) and for lanthanum in the external site (position 2 in Figure 1). The large difference in the atomic number of the two metals was expected to produce a clear distinction of the two centres through X‐ray data.

Indeed, data refinement slightly improves when the metal exchange condition has been introduced in the model (see the X‐ray Diffraction studies section below), highlighting that yttrium atoms are preferentially *O*‐coordinated to pyterpyNO, in the site with a lower coordination number, with convergence being obtained when 91 % of the yttrium atoms are placed in the internal site (position 1), and 9 % in the external one (position 2).

**Figure 2:**
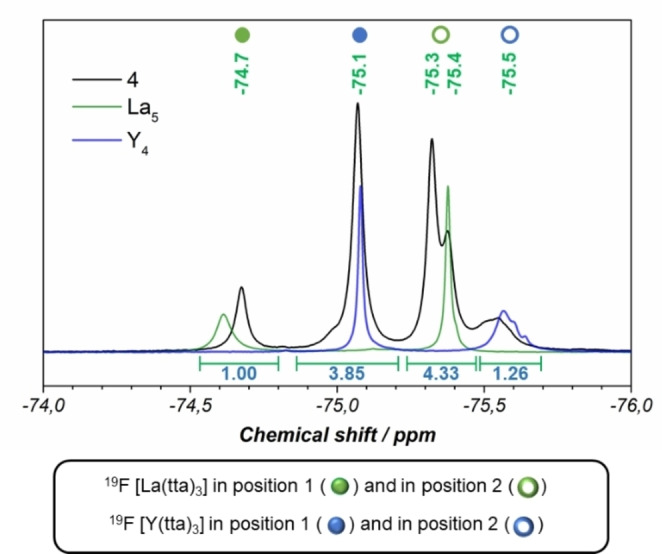
^19^F NMR spectrum of **4** compared with ^19^F NMR spectra of the homometallic compounds of yttrium (Y_4_, in blue) and lanthanum (La_5_, in green).^[70]^

NMR data for the isolated product are consistent with some positional disorder in solution. While ^1^H NMR spectra, with a higher number of larger signals respect to the monometallic compounds,^[70]^ are difficult to use, ^19^F NMR spectra, having a limited number of signals, are more suitable for the discussion. (Figure 2).

Homo‐metallic ^19^F NMR spectra present only two fluorine signals attributable respectively to RE(tta)_3_ fragments *O*‐ and *N*‐coordinated to pyterpyNO,^[70]^ so that signals related to fluorine atoms of the tta diketonato ligands of the hetero‐lanthanide compound can be used to discriminate the two coordination spheres (position 1 or position 2) for the two rare‐earth centres. Chemical shifts at −74.7 and −75.1 ppm can be attributed to tta ligands of *O*‐coordinated lanthanum or yttrium, respectively, while signals at −75.3 and −75.5 ppm can be attributed to *N*‐coordinated lanthanum and yttrium, since they closely resemble the homo‐metallic shifts (Figure 2).^[70]^ Taking into account an estimated error of ±10 % on the integration of the signals, the values obtained can be used for an estimate of the positional disorder in solution. Integrals are consistent with a composition one to one of the two metals with about 79 % of the *O*‐coordinated site (position 1, Scheme 1) occupied by yttrium and about 77 % of the *N*‐coordinated site, (position 2, Scheme 1) occupied by lanthanum. Signals attributable to fluorine atoms bonded to *N*‐coordinated metals are large (even split for lanthanum) and slightly displaced from the position of the homo‐metallic compounds probably due to the presence of multiple slightly different species in solution, since beside positional isomers, a compositional disorder is expected to be operative. For instance, for a Y_2_La_2_(tta)_12_(pyterpyNO)_2_ composition we may have the same number of Y_3_La(tta)_12_(pyterpyNO)_2_ and YLa_3_(tta)_12_(pyterpyNO)_2_ molecules, both having two positional isomers (that is in Y_3_La, La can be *O*‐ or *N*‐coordinated). The major result here is the possibility to give an estimate of the different site occupancy of the two metals in solution to be compared with single crystal X‐ray data. Although a higher positional disorder is present with respect to the solid state study, size selectivity is operative also in solution and no complete scrambling with a statistical distribution occurs. ^89^Y NMR spectra present two signals at 31.4 and at 13.9 ppm due to yttrium centres respectively *O*‐ and *N*‐coordinated to pyterpyNO^[70]^ in an integral ratio of 80/20 in good agreement with ^19^F NMR data. (Figure S6).

The two‐step synthetic protocol has also been used to prepare Dy_2_La_2_(tta)_12_(pyterpyNO)_2_ (**5**) and Eu_2_La_2_(tta)_12_(pyterpyNO)_2_ (**6**), starting from La(tta)_3_(dme) and **3** or **2** for magnetic and luminescence (PL) studies respectively. Both compounds have been crystallized and found to have the same metric of the cell, confirming that the compounds are isotypic and isostructural. X‐ray data have clarified that larger lanthanum centres prefer the external position being coordinated to the terpyridine unit of pyterpyNO, although positional disorder increases as the difference in the ionic radius of the two metals[^73^, ^74^] gets smaller (Shannon data have been here used) and lanthanum occupancy of the *N*‐coordination site decreases from 91 % for **4** to 79 % for **5** and to 70 % for **6**.

While no useful NMR data can be obtained for the dysprosium compound **5**, it was possible to collect a ^19^F NMR of the europium analogue **6** (Figure S3). In this spectrum the fluorine signals are well separated for the two metal centres evidencing the correct molar ratio of the two metals and the presence of a positional disorder. The spectrum could be rationalized with the presence in solution, beside the expected compound, of *O*‐coordinated lanthanum and *N*‐coordinated europium. Here, a positional disorder slightly higher than in the crystallographic studies has been observed, for example the lanthanum occupancy of the *N*‐coordinated site is 63 % for NMR vs. 70 for X‐ray (Table 2). It is worth mentioning that signals at −74.8 and −75.4 ppm closely resemble the values observed for fluorine atoms of *O*‐ and *N*‐coordinated lanthanum in **4** with a higher width (especially the one at −74.8 ppm) possibly due to the presence of the paramagnetic europium centre.

**Scheme 2:**
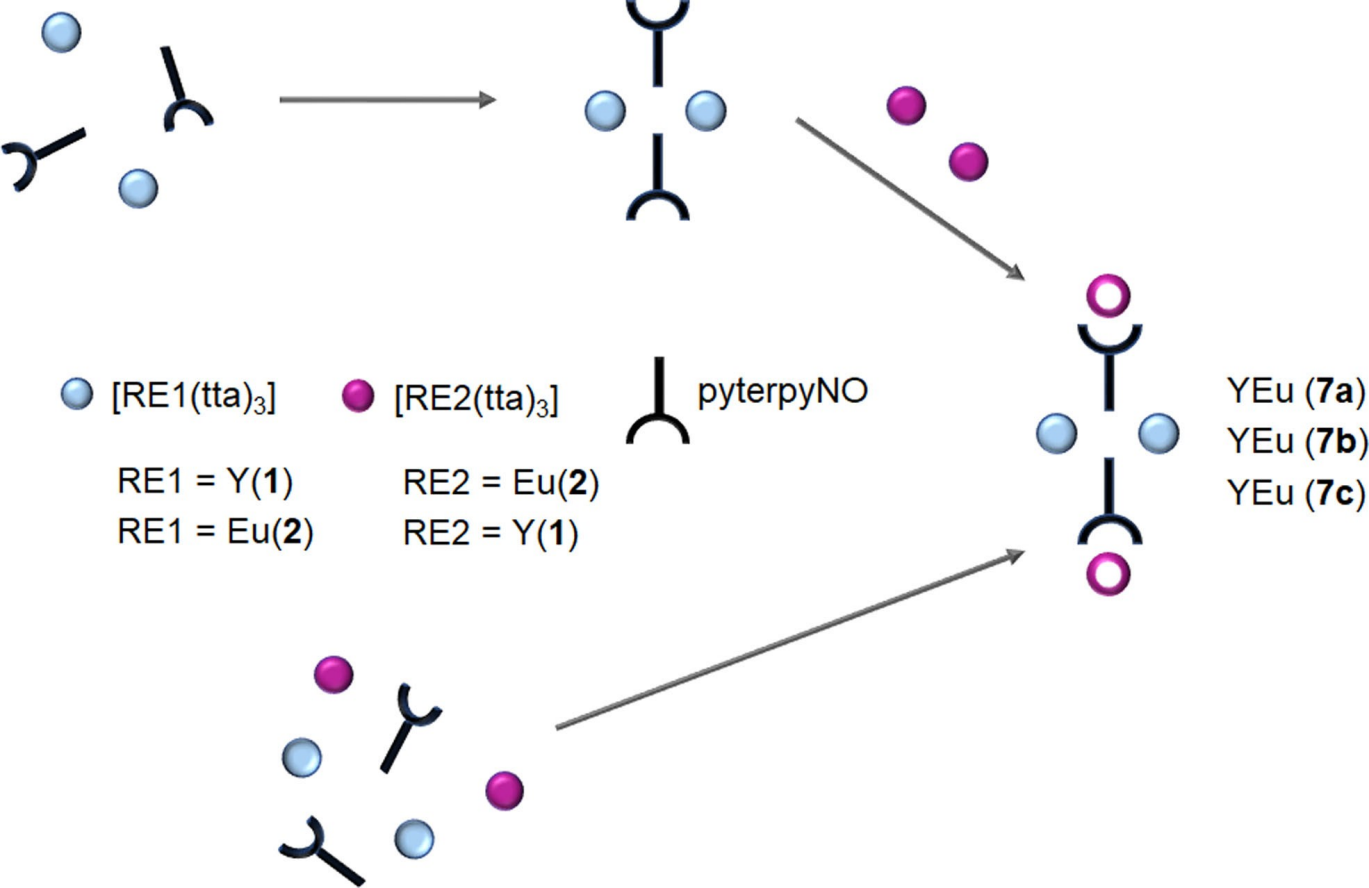
Sequential synthetic approach (**7 a** and **7 b**) and self‐assembly (**7 c**) for the synthesis of **7**.

The two‐step synthetic route for the heterolanthanide (RE1; RE2) complexes, described above, relied on the initial synthesis of the dinuclear complex RE1_2_(tta)_6_(pyterpyNO)_2_ where RE1 is the metal centre having the smaller radius of the two. In the attempt to understand the role of the sequential synthetic approach, the compound with RE1=Y and RE2=Eu has been prepared starting as above from the dinuclear complex **1** and Eu(tta)_3_(dme) (**7 a**, yttrium has a smaller radius) as well as starting from **2** and Y(tta)_3_(dme) (**7 b**) or starting directly from Y(tta)_3_(dme) and Eu(tta)_3_(dme) and pyterpyNO in a single‐step synthesis (**7 c**) Scheme 2.

**Figure 3:**
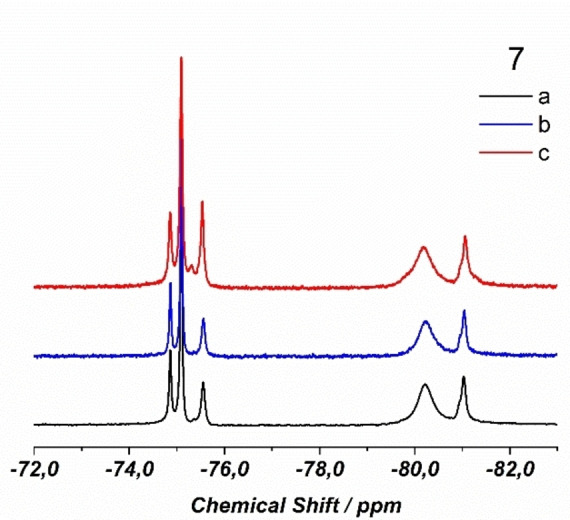
^19^F NMR spectra of the three yttrium europium compounds discussed in the text: **7 a**) starting from **1** and Eu(tta)_3_(dme) (black); **7 b**) starting from **2** and Y(tta)_3_(dme) (blue); **7 c**) starting from Y(tta)_3_(dme) and Eu(tta)_3_(dme) and pyterpyNO (red).

In all examples, reaction occurs at room temperature in toluene and the products, well soluble in toluene, are precipitated with heptane. IR spectroscopy suggests for all experiments the formation of tetranuclear complexes and well‐shaped crystals have been obtained from a toluene solution. X‐ray diffraction studies on single crystals of the three experiments showed the same metric and the same structure: crystals were isotypic with the previous tetranuclear compounds. Significantly, diffraction studies showed that the distribution of the two metals in the two positions is the same in all experiments. Crystallographic studies showed that yttrium atoms (having a smaller radius) mostly occupy the site with coordination number 8 (about 80 % see Table 2). This result shows that positional distribution does not depend on the synthetic protocol and that a self‐assembly reaction yields the same product of a sequential approach. ^19^F NMR studies on the three compounds showed as well almost identical results (Figure 3).

In the spectrum of **7 a** as well as **7 b** or **7 c** (Figure 3 and Figure S5) signals due to yttrium and to europium are well separated. Here, three signals are due to tta bonded to yttrium differently from the spectrum of **4** (yttrium/lanthanum) where only two signals were observed. We suppose that the third signal may be due to an *O*‐coordinated yttrium (position 1) close to a paramagnetic *O*‐coordinated europium centre (position 1). It appears reasonable that a *N*‐coordinated europium centre, is at too large a distance to influence the chemical shift. From the integral values it is possible to estimate a positional disorder close to that found through crystallography (Table 1).

**Table 1:** Size selectivity for compounds **4**–**7**.

Name	Δ*r/* Å	*Pop*.Ln1 %. (X‐ray)	*Pop*. Ln2 % (X‐ray)	*Pop*.Ln1 % (NMR)	*Pop*. Ln2 % (NMR)	*Pop*.Ln1 % (magnetometry)
4	0.141	91.3(2) Y; 8.7(2)La	91.3(2) La; 8.7(2) Y	79(3) Y; 21(3) La	77(3) La; 23(3) Y	
5	0.133	78.9(3) Dy; 21.1(3) La	78.9(3) La; 21.1(3) Dy			80(3) Dy; 20(1) La
6	0.096	69.9(5) Eu; 30.1(5) La	69.9(5) La; 30.1(5) Eu	56(5) Eu; 44(5) La	63(5) La; 37(5) Eu	
7a	0.045	78.3(2) Y; 21.7(2) Eu	78.3(2) Eu; 21.7(2) Y	71(3) Y; 29(3) Eu	77(3) Eu; 23(3) Y;	
7b	0.045	77.0(2) Y; 23.0(2) Eu	77.0(2) Eu; 23.0(2) Y	70(4) Y; 30(4) Eu	78(4) Eu; 22(4) Y	
7c	0.045	76.5(2) Y; 23.5(2) Eu	76.5(2) Eu; 23.5(2) Y	71(4) Y; 29(4) Eu	70(4) Eu; 30(4) Y	

## 3 Magnetic study of 5

**Figure 4:**
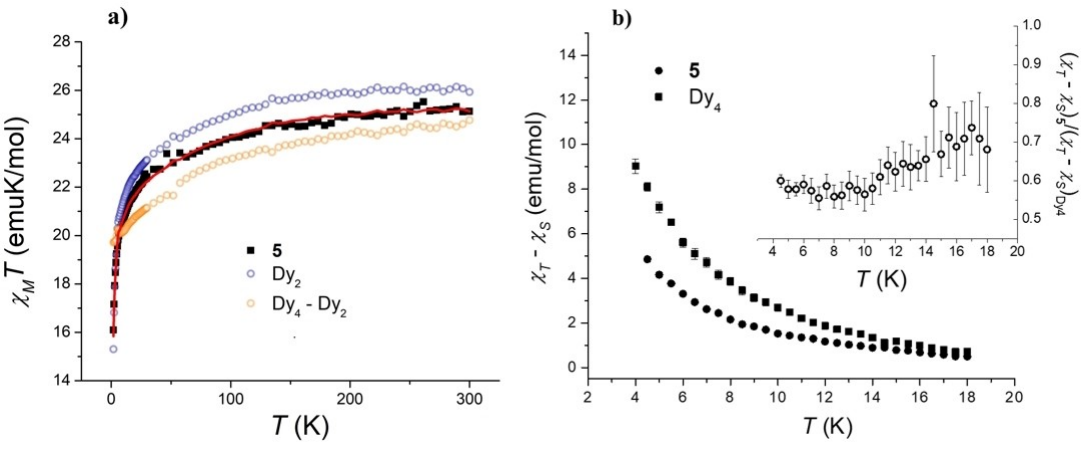
*Panel a*: Temperature dependence of the *χ_M_T* product for compounds **5**, Dy_2_, and difference of the *χ_M_T* products of Dy_4_ and Dy_2_. The red line is the result of the linear combination of Dy_2_ and Dy_4_‐Dy_2_ χ_*M*_ *T* plots used to reproduce the behaviour of **5**, as described in the text. *Panel b*: Temperature dependence of the difference between the isothermal (χ_T_) and adiabatic (χ_S_) magnetic susceptibilities of **5** and its homo‐metallic analogue Dy_4_ measured with 1 kOe field applied. The inset displays the ratio between the two sets of values.

The temperature dependence of the χ_M_T product of compound **5** is reported in Figure 4. The experimental room temperature χ_M_T value (25.13 emuK/mol) is lower than the one expected for two free Dy(III) ions (28.33 emuK/mol), as frequently found for Dy(III) complexes. Upon cooling, this value lowers to reach 16.09 emuK/mol at 1.8 K, due to the splitting of the ^6^H_15/2_ electronic ground multiplet of the ion by means of the crystal field^[75]^ and to the presence of the antiferromagnetic interaction between the two Dy(III) ions bridged by the oxygens of the two NO groups in the unscrambled **5** molecule. The isothermal magnetizations, measured at 1.9 and 4.0 K, display a saturation value of 9.27 μ_B_/mol (Figure S7). Both values are in line with what reported for the dinuclear [Dy_2_(tta)_6_(pyterpyNO)_2_] (Dy_2_) complex.^[70]^

The temperature evolution of the χ_*M*_ *T* product depends on the splitting of the ground electronic multiplet of the rare earth ion induced by the ligand field, and it is thus expected to change when the Dy^3+^ ion is coordinated to the position 1 or position 2, whose geometrical parameters, including the coordination number, are different. In addition, Dy^3+^ ions in position 1 are known to interact antiferromagnetically,^[70]^ further differentiating their magnetic properties from the ones of the outer, non‐interacting, ions in position 2. In order to obtain information about the positional disorder between the Dy^3+^ and La^3+^ ions in compound **5**, the temperature dependence of the χ_*M*_ *T* of compound **5** has been fitted using a linear combination of the χ_*M*_ *T* plot of compounds Dy_2_ and [Dy_4_(tta)_12_(pyterpyNO)_2_] (Dy_4_)^70^ (Figure 4), according to Equation 1:

(1) $$\chi {}_{M}T\;\left(5\right)=\alpha \;\cdot \;\chi {}_{M}T\;\left(\mathrm{Dy}{}_{2}\right)+\beta \;\cdot \;\left[\chi {}_{M}T\;\left(\mathrm{Dy}{}_{4}\right)-\chi {}_{M}T\;\left(\mathrm{Dy}{}_{2}\right)\right]$$

where α and β are the numerical coefficients of the linear combination, used as fitting parameters.

The Dy_4_ complex is the homometallic analogue of complex **5**, featuring four dysprosium(III) ions, split between positions 1 and 2. Dy_2_, on the other hand, features only two dysprosium(III) ions located in position 1. Notably, the coordination environment around the two Dy^3+^ ions in position 1 in Dy_4_ is almost superimposable with the one found for the two Y^3+^ ions in Y_2_ (isostructural diamagnetic analogue of the Dy_2_ system, Figure S8 and Table S1). As a result, the χ_*M*_ *T* product of the Dy_2_ system is expected to reproduce the behaviour of the Dy ions in position 1 in **5**, while the contribution of their counterpart in position 2 can be estimated through the difference in the χ_*M*_ *T* values of Dy_4_ and Dy_2_. This approach holds on the basis of the closeness in structure of the first coordination sphere of the dysprosium(III) ions in position 1 in compounds Dy_2_ and Dy_4_.^1^ Considering a molar fraction of Dy^3+^ ions in position 1 of 0.80(3), and consequently a molar fraction of 0.20(1) in position 2, it is possible to reproduce the χ_*M*_ *T* of **5** using Equation (1) with a high degree of accuracy (α=0.78(1), β=0.20(2), R^2^=0.99995). The results of this procedure are in close agreement with the molar fractions of Dy^3+^ ions in position 1 and 2 obtained from the crystallographic analysis of compound **5**. To the best of our knowledge, an approach involving the magnetic properties of molecular analogues has never been used to determine the positional disorder, or degree of scrambling, of two different ions between two non‐equivalent coordination positions.

The dynamics of the magnetization of compound **5** are reported in Figure S9 as temperature dependence of the in‐phase and out‐of‐phase magnetic susceptibilities measured without static field applied for eleven sweeping frequencies of the oscillating field. As previously observed for the homometallic Dy_4_(tta)_12_(pyterpyNO)_2_ analogue of **5**,^[70]^ two sets of peaks appear in the out‐of‐phase susceptibility, which move to higher temperature upon increase in sweeping frequency, highlighting a Single Molecule Magnet behaviour. A fitting procedure of these peaks using an extended Debye model has been carried out (Figure S9); however, due to the high uncertainty in the determination of the magnetic relaxation times of the faster process, only the relaxation times of the slower one have been taken in consideration in the following discussion, and their temperature dependence is reported in Figure S10. Again, similarly to its homo‐metallic Dy_4_ analogue, the best model to fit the data has been a Raman/direct mixed relaxation process (*n*=6.1(1)), where the use of the direct process is justified in zero field due to the presence of the magnetic coupling between the two Dy(III) ions, which leads to a pair of weakly split levels and at the same time reduces the tunneling of the magnetization. For this reason, the 1 kOe dynamics are almost superimposable to the zero‐field data, showing a slight increase in the low temperature relaxation rate, and has been fitted with the same model mentioned above (*n*=4.7(2)). Static fields applied spanning the 0–2 kOe range showed almost no effect on the relaxation times at 5.0 K (Figure S11), the main effect being an overall increase in the slow relaxing fraction of the magnetization, as can be seen by the increase of the out‐of‐phase part of the magnetic susceptibility for the 1 kOe data.

Due to the close similarity of the relaxation behaviour of **5** with that of its homometallic Dy_4_ counterpart (Figures S10 and S12), we have plotted the ratio between the difference of the isothermal (χ_*T*_) and adiabatic (χ_*S*_) magnetic susceptibilities of **5** and Dy_4_, measured in a 1 kOe static field (inset of panel b of Figure 4), as a tentative approach to correlate positional disorder and slow relaxing molar fraction in **5**. The difference between χ_*T*_ and χ_*S*_ is indeed proportional to the magnetic moment which is relaxing slowly on our instrumental time scale. In the absence of positional disorder one would expect a χ_*T*_‐χ_*S*_ temperature dependence superimposable for the two systems. It must be stressed that this approach relies on a large set of assumptions and should be taken as a complementary technique to the X‐ray diffractometry and DC magnetometry data. First, we assume that, for both molecules, the slow relaxing magnetic moment arises from the dinuclear Dy_2_ core, featuring Dy^3+^ ions with an antiferromagnetic exchange channelled through the two bridging oxygen atoms, and not from the nona‐coordinated Dy^3+^ ions located in position 2. Such an assumption is supported by the slight decrease in the magnetic relaxation time upon passing from zero to 1 kOe static field applied (Figure S11), a phenomenon typical of coupled dysprosium(III) Single Molecule Magnets and, as such, observed also for Dy_4_,^[70]^ while it is unusual for uncoupled Dy^3+^ Single Ion Magnets. Moreover, this treatment relies on the assumption that the dipolar field acting on the slow relaxing Dy_2_ core is the same for **5** and Dy_4_. This is an oversimplification, due to the different concentration of paramagnetic ions in the two systems; anyway, we can expect the importance of this effect to be lowered, since we are comparing AC data taken with an applied static field of 1 kOe. That said, the results reported in the inset of the b panel of Figure S10 display a ratio moving from 0.6 in the 4.5–10 K range to 0.7 reaching 18.0 K. Considering the sensitivity of the magnetic relaxation features in molecular systems, an almost constant χ_*T*_–χ_*S*_ ratio is not an obvious result, and points out a very similar dynamics of the magnetization in **5** and Dy_4_, supporting the validity of our method. This approach yielded an estimation of a positional disorder where about 30–40 % of dysprosium atoms are *N*‐coordinated in position 2 (Scheme 1), with a 10–20 % discrepancy in comparison with the results of X‐ray diffraction and magnetometry. Such a difference can be ascribed to the subtle effects governing the relaxation times and the slow relaxing molar fractions in molecular materials.

## 4 Luminescent properties of 6 and 7

Single crystal X‐ray diffraction, magnetism, and NMR studies showed the presence of size selectivity in the occupancy of octa‐ (CN8) and nona‐coordinate (CN9) sites both in the solid state and in solution. We planned to use the information on the occupancy of the two sites, obtained so far via NMR and single crystal XRD, to shed some light on the PL of the hetero‐bimetallic complexes. For this reason, PL experiments were conducted on millimolar solutions of complexes **6**, **7 a**, **7 b**, **7 c** in toluene, to keep the experimental conditions as close as possible to those employed for NMR analyses, leading to a model for analysing the emission spectra and to get information about the two sites.

In a previous study on di‐ and tetra‐nuclear homo‐metallic Eu^3+^ complexes with the pyterpyNO ligand, we showed that the Eu^3+^‐centred emissions are mainly determined by the tta ligands.^[70]^ Though the high chemical similarity between the two sites makes an unambiguous distinction of the two contributions nontrivial, small differences in the emissions of europium occupying CN8 and CN9 sites were pointed out (Figure S13). Moreover, the unavailability of a complex where europium occupies only the CN9 sites complicates the study of photoluminescence of these complexes because of the severe spectral overlaps.

**Figure 5:**
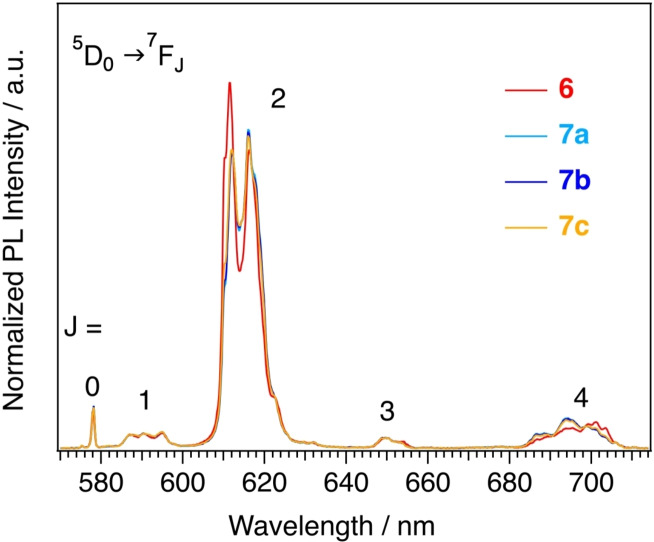
Photoluminescence spectra of millimolar solutions of **6**, **7 a**, **7 b**, and **7 c** in toluene. The spectra were collected exciting the samples at 370 nm (maximum of the excitation spectra). Only europium emissions were detected.

The emission spectra of the hetero‐bimetallic tetranuclear complexes (**6**, **7 a**, **7 b**, **7 c**, Figure 5) are quite similar, presenting however small differences in the ^5^D_0_→^7^F_2_ (600–630 nm) and ^5^D_0_→^7^F_4_ (680–710 nm) transitions. Similar considerations apply when these spectra are compared with those of the homo‐metallic complexes **2** and Eu_4_(tta)_12_(pyterpyNO)_2_ (Figure S13).

Since the millimolar concentrations used for NMR consistency are outside the standard range in spectroscopic studies, it was necessary to develop an interpretative model to get information from the emission spectra of europium complexes. The same brightness for the emissions of Eu^3+^ in CN8 and CN9 sites was initially assumed on the base of the marked chemical similarity between these two sites. According to this hypothesis, equal integrated areas of their emission spectra were expected for two equally occupied sites. Therefore, the following relations were assumed:

(2) $$A{}_{\mathrm{CN}8}=A{}_{\mathrm{CN}9}=1$$

(3) $$A{}_{\mathrm{Eu}4}=A{}_{\mathrm{CN}8}+A{}_{\mathrm{CN}9}=2$$

where A_CN8_, A_CN9_, and A_Eu4_ are the integrated areas of the emission spectra of sites having coordination number 8 (**2**), 9 (non‐available species), and of the previously characterized homonuclear tetranuclear Eu_4_(tta)_12_(pyterpyNO)_2_, respectively. Using Equation (2) and Equation (3) it is possible to derive the spectrum of the pure CN9 species and use it to simulate the spectra of the compounds **6**, **7 a**, **7 b**, **7 c** as linear combinations of the spectra of the two sites. The information on site occupancy is carried by the coefficients of the linear combinations.

**Figure 6:**
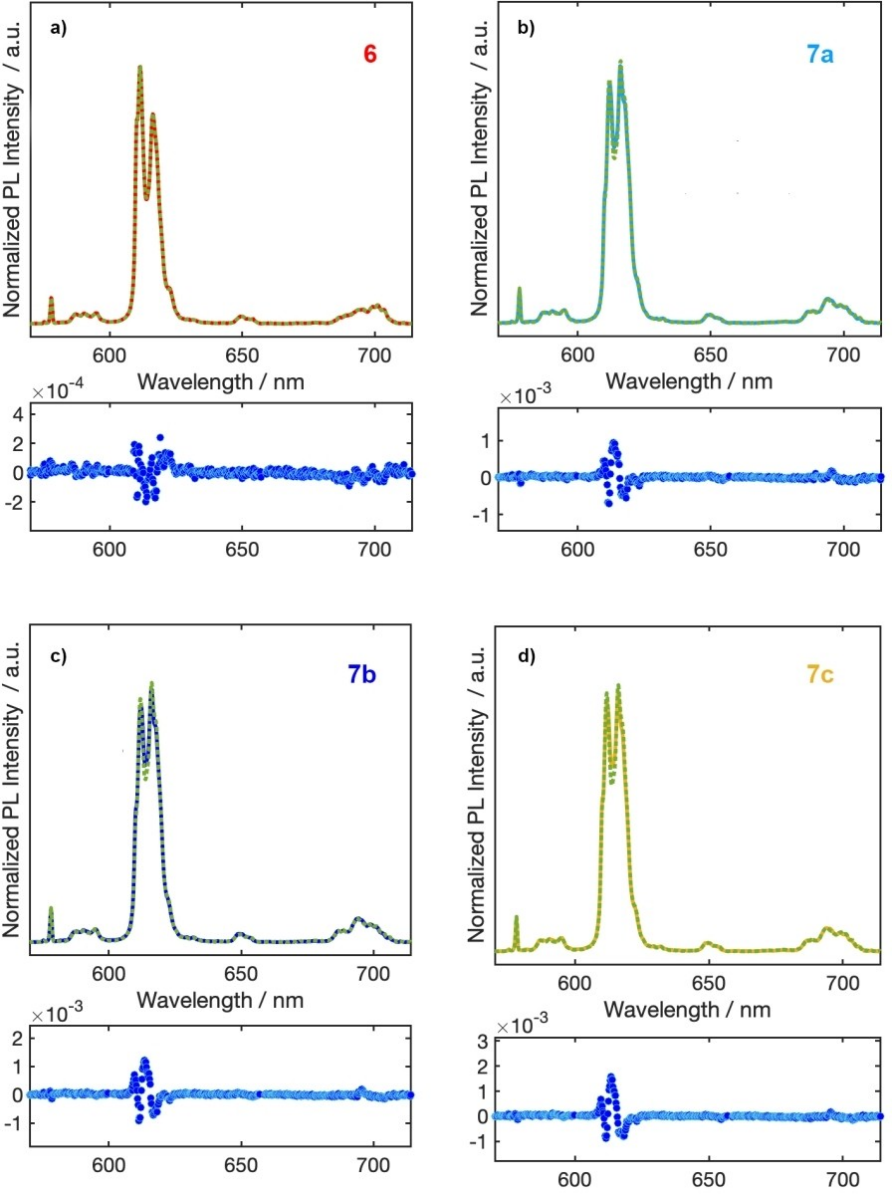
Comparison between experimental (continuous line) and calculated (dotted line) spectra for **a) 6**, **b) 7 a**, **c) 7 b**, and **d) 7 c**.

Looking at the residuals and **χ** ^2^ values, close to one for all the samples, the simulations (Figure 6) were already quite satisfactory. From this analysis, the europium occupancy of the CN8 site was about 65 % for **6**, ca. 5 % for **7 a** and **7 b**, and only slightly higher for **7 c** (around 10 %). With the assumptions made so far, results in agreement with NMR and diffraction data were obtained for **6**, but CN8 site occupancy for **7 a**–**c** was underestimated so that an improved model was necessary.

Our previous studies on dinuclear europium β‐diketonato complexes having 4,4’‐bipyridine *N*‐oxide or pyrazine *N*‐oxide as *O*‐donor bridging ligands have shown that, despite their very similar structure, the absolute emission quantum yields and thermometric properties markedly changed by modifying the *N*‐oxide ligand.^[69]^ We also highlighted the overriding role of charge transfer transitions in determining these behaviours. Based on these evidences, the emission profile of Eu^3+^ located in the CN9 site was evaluated introducing a correction coefficient *n* to take into account the different brightness for the two sites (Eq. 4).

(4) $$A{}_{\mathrm{CN}8}=n\;A{}_{\mathrm{CN}9}$$

The parameter *n* was varied in the range 0.67–9 and the emission spectra of compounds **6**, **7 a**, **7 b**, **7 c** were calculated, as before, as linear combinations of the spectra of the species at CN8 and CN9.

**Figure 7:**
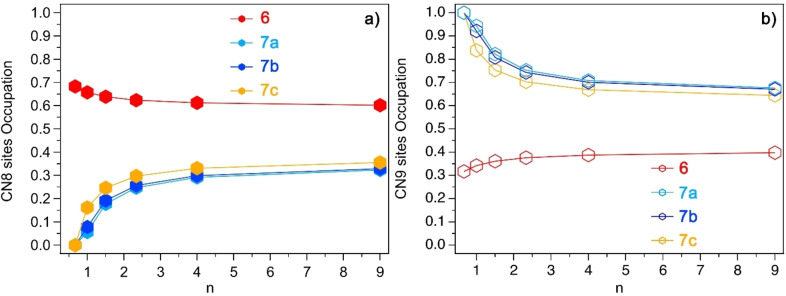
Occupancy of CN8 a) and CN9 b) sites as a function of the parameter n.

The trend in the values of the coefficients of the linear combinations as a function of parameter *n* is shown in the Figure 7.

The emissions of complex **6** are relatively insensitive to the variation of the parameter, and the results of the analysis of the spectra show the presence of a disorder in the occupancy of the two sites of about 30 %, in line with what is shown by NMR and XRD. For complexes **7 a**–**c**, however, the majority of Eu^3+^ ions occupy sites with coordination number 9 and the emissions are instead very sensitive to the variation of n, i. e. to the difference in brightness between the two sites. In this case, the occupancy of CN8 site goes from 0 % for n=0.67 to 30 % for *n*=4. The trend for complexes **7 a**, **7 b** and **7 c** is analogous. For both **6** and **7 a**–**c** complexes the best agreement with NMR and diffraction data is obtained for *n* values around 2.

According to the optimized analytical model, the two sites have a considerable difference in brightness and in particular the site at CN9 turns out to be more quenched than that at CN8. We have already observed similar differences in homonuclear dinuclear complexes of general formula [Eu_2_(β‐diketonato)_6_(N‐oxide)_y_] for which the different behaviour was attributed to different contributions of charge transfer transitions.^[69]^ An important contribution from CTs is plausible in this case as well. The combined use of NMR and PL spectroscopies has allowed us to obtain important information on a system intrinsically complicated to study because of the heavy spectral overlap.

## 5 Discussion

As reported in Table 1, a statistical distribution of the two metal cations in the two different positions of the tetranuclear compounds is not present for all the analysed compounds, the smaller ion being preferentially located in the position 1. Size selectivity can be therefore used to rationalize the general trend of our data; for lanthanum containing compounds the difference in the ionic radius (Y, Δ*r*=0.141 Å (**4**); Dy Δ*r*=0.133 Å (**5**); and Eu Δ*r*=0.096 Å (**6**)) is decreasing as well as the *N*‐coordinated lanthanum percentage (91; 79; 70 % respectively from crystallography). For Y containing compounds, a similar trend is observed for the occupancy of yttrium in position 1: for compounds **4** and **7** (Δ*r*=0.141 Å and 0.045 Å) the *O*‐coordinated yttrium occupancy (from crystallography) decreases from 91 to 77 %. In compounds **6** and **7**, europium atoms show a preference to be *O*‐coordinated and *N*‐coordinated, respectively, having a smaller ionic radius than lanthanum but larger than yttrium.

Nevertheless, positional disorder seems to be not exclusively related to size and a slightly higher metal positional disorder (X‐ray and NMR) for the Eu/La derivative (Δ*r* 0.096 Å) respect to the Y/Eu analogue (Δ*r* is 0.045 Å) is possibly due to a different contribution. This result is unusual since rare‐earth centres share a prevalently electrostatic chemistry where the ionic radius is the major source of differentiation. A possible minor contribution may be found in the competitive interaction of two rare‐earth centres with the *N*‐oxide and the terpyridine moiety. It is well known that rare‐earth metals are all oxophilic centres showing stronger RE−O interactions going to the right of the series. At the same time, the softer donor character of the terpyridine unit is demonstrated by its higher affinity towards actinide(III) ions when compared to lanthanides.[^76^, ^77^, ^78^, ^79^] Usually, this difference is ascribed to an increased tendency to form covalent bonds between the An(III), featuring diffuse and less shielded 5 f orbitals, when compared with the highly shielded 4f ones. Several literature data, mostly obtained from rare‐earth separation processes, show for the *pseudo*‐lanthanide yttrium, having a ionic radius close to holmium, an “itinerant” behaviour across the lanthanide series, with respect to the extraction system. In particular, yttrium resembles heavy lanthanides for interaction with very electronegative donors (F or O donors) but behaves as an early lanthanide centre for interaction with less electronegative donors (N donors).[^80^, ^81^, ^82^, ^83^, ^84^, ^85^, ^86^] Moreover, in the literature it has been reported that the stability constants of [Ln(terpy)_***n***_]^**3**+^ increase progressively across the lanthanide series,^[87]^ while a deviation from the expected electrostatic trend has been observed for the affinity of the Ln(hfa)_3_ fragment towards a tridentate nitrogen donor (*NNN*) ligand resulting in a bell‐shaped trend with a maximum for middle‐sized lanthanides.^[88]^ Indeed, it has been noted for the Y(tta)_3_ fragment a weaker bonding interaction towards a tridentate nitrogen donor ligand respect to Eu(tta)_3_.^[89]^ In this respect, it appears reasonable that in **7**, yttrium atoms, for their smaller size prefer *O*‐coordination more than europium atoms while they show a lower affinity towards *N*‐donors respect to europium centres. Both contributions work in the same direction, reducing the positional disorder for the Y/Eu derivative respect to the Eu/La analogue where the higher affinity of the europium fragment for the terpyridine moiety respect to lanthanum could slightly disfavour the expected distribution. As a result, the electrostatic trend based only on the difference in ionic radius is not strictly respected. Moreover, also the small positional disorder encountered for the Y/La derivative, respect to the Dy/La analogue is possibly related not only to the difference in the ionic radius between the two metal centres but also to the relatively scarce affinity of yttrium with respect to dysprosium for nitrogen donors.

## 6 Conclusions

Tetranuclear centrosymmetric lanthanide complexes RE1_2_RE2_2_(tta)_12_(pyterpyNO)_2_ presenting two distinct metal positions with different coordination environments have been prepared (RE1/RE2=Y/La, **4**, Dy/La, **5**, Eu/La, **6**) in a two‐step synthesis, where the initially formed dinuclear complex of the smaller cation RE1_2_(tta)_6_(pyterpyNO)_2_ (RE1=Y, Dy, Eu) was reacted with two equivalents of La(tta)_3_dme in toluene at room temperature. All the complexes are isostructural, the smaller RE1 ion being preferentially coordinated by the *O*‐donor atoms of the pyterpyNO ligand, and the larger bound by the terpyridine moiety, with no statistical distribution between the two sites. ^19^F NMR, single crystal X‐ray diffraction, alternated‐current susceptibility and photoluminescence have been used to evaluate the size selectivity in solution and in the solid state. In **4**, **5** and **6**, lanthanum occupancy of the nona‐coordinated site decreases (from 91 to 79 to 70 %) consistently with the progressively decreasing difference in the ionic radii. The synthesis of the mixed Eu/Y compound, **7**, obtained reacting **1** with two equivalents of Eu(tta)_3_dme, **7 a**, but also reversing the sequential protocol, **7 b**, or using a self‐assembly route, **7 c**, has evidenced that the nature of the product does not depend on the adopted synthetic protocol, in line with the kinetic lability of lanthanide ions. Remarkably single crystals X‐ray and NMR studies for the three compounds **7 a** **c** have shown the same lanthanide distribution over the two sites of the tetranuclear complex. In addition, DC magnetometry has been used for the first time to quantify intramolecular ionic distribution in the solid state. Furthermore, it appeared very interesting to notice for **7** a lower positional disorder than expected, considering the small difference in the ionic radii of the two centres, suggesting that lanthanide distribution is not only due to size selectivity in this case. The discrepancy has been accounted for considering the “itinerant” behaviour of yttrium in the lanthanide series and the higher affinity of β‐diketonato complexes of Eu(III) towards the terpyridine unit. Present results are relevant since they show that selectivity is not exclusively governed by simple electrostatic interactions. Finally, the combined use of ^19^F NMR and photoluminescence allowed us to obtain information on the behaviour of two Eu^3+^ sites intrinsically difficult to study due to the strong spectroscopic overlap. We found that *N*‐ and *O*‐coordinated pyterpyNO ligands quench europium emission to a different extent, i. e. the *O*‐coordinated site is the brighter. Taken together, the present results highlight the potential of the simple ditopic ligand pyterpyNO for preparing ordered heterolanthanide molecular systems, opening the way for the investigation of structural and chemical effects on the size selectivity in this class of materials. Moreover, access to simple hetero‐lanthanide functional structures, efficient in the chemical recognition process, may give an easy entry into bifunctional probes featuring magnetic and luminescent properties.

## Experimental Section

**Materials and characterizations**: Anhydrous solvents were purchased from Merck and used as received. 4’‐(4‐pyridil)‐2,2’:6’,2”‐terpyridine *N*‐oxide (pyterpyNO) was synthesized according to the literature.^[90]^ RE(tta)_3_(dme) (RE=Y, Dy, Eu and La) and RE_2_(tta)_6_(pyterpyNO)_2_ (RE=Y, Dy and Eu) have been prepared as previously reported.^[70]^ FTIR spectra on solid samples were recorded with a Perkin–Elmer “Spectrum One” spectrometer, equipped with an ATR accessory. ^19^F NMR spectra were recorded with a Bruker “Avance DRX400” spectrometer. Chemical shifts were measured in ppm (δ) from CFCl_3_ for ^19^F. Elemental analysis (C, H, N) was performed using an Elementar “vario MICRO cube” instrument, at Dipartimento di Chimica e Chimica Industriale, Università di Pisa (Italy).

Room temperature luminescence spectra were recorded with a Horiba JobinYvon *Fluorolog‐3* spectrofluorimeter. A full description of the employed set‐up can be found in a previous paper.[^70^, ^91^] Linear combinations of the emission spectra and the determination of site occupancy were performed with Matlab 2021b using the Optimization Toolbox. For the calculations the integrated areas of the emission spectra of all complexes but Eu_4_(tta)_12_(pyterpyNO)_2_ were normalized. The integrated area of Eu_4_(tta)_12_(pyterpyNO)_2_ PL spectrum was set equal to 2.

**Magnetization measurements**: Samples employed for DC (direct current) and AC (alternating current) measurements consisted of pressed microcrystalline powders of **5**, wrapped in Teflon(TM) tape. The DC magnetic characterization was performed on Quantum Design MPMS (Magnetic Properties Measurement System) equipment provided with a 5 T magnet. The magnetization (*M*) dependence with the absolute temperature was investigated between 300 and 55 K using a magnetic field (*B*) of 10 kOe, and between 55 and 2 K with a field of 1 kOe to prevent magnetic saturation. Magnetic susceptibility per mole (*χ_M_*) was then evaluated as *χ_M_* =*M_M_*/*B*.

The molar fractions of Dy^3+^ ions in position 1 and 2 have been calculated from the fitting of the temperature dependence of the *χ_M_T* product using Equation (1), as reported in the main text, using the following normalizing equations:

$$\mathrm{Dy}\left(\mathrm{III}\right){}_{1}=\alpha \;{}^{*}\;1/\left(0.78+0.20\right)$$

$$\mathrm{Dy}\left(\mathrm{III}\right){}_{2}=\beta \;{}^{*}\;1/\left(0.78+0.20\right)$$

Alternating current magnetic susceptibility analyses were performed with a PPMS (Physical Properties Measurement System) platform, also from Quantum Design, with oscillating field frequencies ranging from 10 to 10^4^ Hz, and using static magnetic fields of zero and 1 kOe. The resulting magnetic data were corrected for the diamagnetic contributions of the ligands calculated from Pascal constants,^[92]^ together with those measured for the sample container and the wrapping Teflon tape.

The ac susceptibility data were analysed within the extended Debye model,[^93^, ^94^] in which a maximum in the out‐of‐phase component χ_*M*_ *′′* of the complex susceptibility is observed when the relaxation time τ equals (2πυ)^−1^. The adopted model includes two different relaxation processes to reproduce a non‐zero χ_*M*_ *′′* in the high frequency region of the plots; the corresponding relaxation times extracted from this component were however not described due to the huge uncertainty associated to them. The frequency dependence of χ_*M*_ *′′* at constant temperature was thus fitted using Equation 5:

(5) $$\chi {}_{M}{}^{''}\;\left(\tau \right)=\chi {}_{M}{}^{''}{}_{FIT}\;\left(\omega \right)+\chi {}_{M}{}^{''}{}_{HF}\;\left(\omega \right)$$

where each process corresponds to the function reported in Equation 6:

(6) $$\chi {}_{M}{}^{''}\;\left(\omega \right)=\left(\chi {}_{T}\;\text{-}\;\chi {}_{S}\right)\left[\left(\omega \tau \right){}^{1-\alpha }\cos\left(\alpha \pi /2\right)\right]/[1+2\left(\omega \tau \right){}^{1-\alpha }\sin\left(\alpha \pi /2\right)+\left(\omega \tau \right){}^{2-2\alpha }].$$

Here ω=2πυ, χ_*T*_ and χ_*S*_ are the isothermal and adiabatic susceptibilities, i. e., the susceptibilities observed in the two limiting cases υ→0 and υ→∞, respectively, and α is a parameter which accounts for a distribute on of relaxation times. In order to reduce overparametrization, the α parameter has been kept the same for the low and high frequency peaks.

The temperature dependence of the magnetic relaxation times τ has been fitted using Equation 7:

(7) $$\tau^{-1}\left(T\right)=CT^{n}+BT$$

where the terms represent a Raman and direct relaxation mechanisms, respectively.

## Syntheses

**Y_2_La_2_(tta)_12_(pyterpyNO)_2_**, **4**: PyterpyNO (0.08 g; 0.25 mmol) and Y(tta)_3_(dme) (0.21 g; 0.25 mmol) were suspended in toluene (12 mL). After few minutes, we obtained a solution, which was cooled down to RT. La(tta)_3_(dme) (0.22 g; 0.25 mmol) was then added and the resulting solution was vigorously stirred for 3 h. Upon adding heptane (50 mL) precipitation of a colourless solid occurred; this solid was then filtered and dried under vacuum for 8 h. We recovered 0.35 g of Y_2_La_2_(tta)_12_(pyterpyNO)_2_ (0.09 mmol, yield 75.7 %). Elem. Anal. found (calcd) for C_136_H_76_F_36_La_2_N_8_O_26_S_12_Y_2_ (%)::C, 43.7 (43.4); H, 2.2 (2.0); N, 3.0 (3.0); S, 10.7 (10.2). IR (range 1700–1100 cm^−1^): 1603 s, 1576w, 1536 s, 1505 m, 1471w, 1412 s, 1355w, 1298 s, 1244 m, 1229 s, 1184 s, 1133 s. Slow evaporation of a toluene solution yielded well‐shaped, single crystals, suitable for X‐ray analysis. Crystals collapse loosing solvent when dried in vacuo.

**Dy_2_La_2_(tta)_12_(pyterpyNO)_2_**, **5**: PyterpyNO (0.12 g; 0.37 mmol) and Dy(tta)_3_(dme) (0.33 g; 0.35 mmol) were dissolved in toluene (12 mL). After few min, to a light yellow solution La(tta)_3_(dme) (0.32 g; 0.36 mmol) was added and the resulting light orange solution was vigorously stirred for 3 h. Upon adding heptane (25 mL) precipitation of a colourless solid occurred; this solid was then filtered and dried under vacuum for 8 h. We recovered 0.42 g of Dy_2_La_2_(tta)_12_(pyterpyNO)_2_ (0.11 mmol, yield 60.3 %). Elem. Anal. found (calcd) for C_136_H_76_N_8_Dy_2_F_36_La_2_O_26_S_12_ (%): C, 41.5 (41.8); H, 2.0 (2.0); N, 2.8 (2.9); S, 10.1 (9.8). IR (range 1700–1100 cm^−1^): 1601 s, 1536 m, 1504 m, 1470 m, 1412 m, 1354w, 1297 s, 1244w, 1229 m, 1183 m, 1133 s. Slow evaporation of a toluene solution yielded well‐shaped, single crystals, suitable for X‐ray analysis. Crystals collapse loosing solvent when dried in vacuo.

**Eu_2_La_2_(tta)_12_(pyterpyNO)_2_**, **6**: PyterpyNO (0.13 g; 0.40 mmol) and Eu(tta)_3_(dme) (0.36 g; 0.40 mmol) were dissolved in toluene (20 mL). After few min, to a light yellow solution La(tta)_3_(dme) (0.36 g; 0.40 mmol) was added and the resulting solution (no change of color was noticed) was vigorously stirred for 3 h. Upon adding heptane (25 mL) precipitation of a colourless solid occurred that was filtered and dried under vacuum for 8 h. We recovered 0.59 g of Eu_2_La_2_(tta)_12_(pyterpyNO)_2_ (0.15 mmol, yield 75.6 %). Elem. Anal. found (calcd) for C_136_H_76_N_8_Eu_2_F_36_La_2_O_26_S_12_ (%): C, 42.1 (42.0); H, 2.0 (2.0); N, 2.8 (2.9); S, 10.2 (9.9). IR (range 1700–1100 cm^−1^): 1601s, 1578w, 1536m, 1505w, 1470m, 1412m, 1355w, 1298s, 1244w, 1229m, 1182m, 1139s. Slow evaporation of a toluene solution yielded well‐shaped, single crystals, suitable for X‐ray analysis. Crystals collapse loosing solvent when dried in vacuo.

**Eu_2_Y_2_(tta)_12_(pyterpyNO)_2_**, **7 a**.) PyterpyNO (0.089 g; 0.28 mmol) and Y(tta)_3_(dme) (0.23 g; 0.28 mmol) were dissolved in toluene (15 mL). After few min, a light yellow solution Eu(tta)_3_(dme) (0.25 g; 0.28 mmol) was added and the resulting solution (no change of colour was noticed) was vigorously stirred for 1 h. Upon adding heptane (50 mL) precipitation of a colourless solid occurred that was filtered and dried under vacuum for 8 h. We recovered 0.44 g of Y_2_Eu_2_(tta)_12_(pyterpyNO)_2_ (0.19 mmol, yield 83.9 %). Elem. Anal. found (calcd) for C_136_H_76_N_8_Y_2_F_36_Eu_2_O_26_S_12_ (%): C, 43.3 (43.1); H, 2.0 (2.0); N, 2.9 (3.0); S, 9.9 (10.2). IR (range 1700–1100 cm^−1^) 1600f, 1536m, 1502d, 1474d, 1412f, 1355d, 1299f, 1247m, 1229m, 1182m, 1130f. Pentane vapour diffusion in a toluene solution of the product yielded well‐shaped, single crystals, suitable for X‐ray analysis. Crystals collapse loosing solvent when dried in vacuo.

7b) PyterpyNO (0.09 g; 0.28 mmol) and Eu(tta)_3_(dme) (0.25 g; 0.28 mmol) were dissolved in toluene (20 mL). After few min, a light yellow solution, Y(tta)_3_(dme) (0.24 g; 0.28 mmol) was added and the resulting solution was vigorously stirred for 3 h. Upon adding heptane (25 mL) precipitation of a colourless solid occurred that was filtered and dried under vacuum for 8 h. We recovered 0.22 g of Y_2_Eu_2_(tta)_12_(pyterpyNO)_2_ (0.12 mmol, yield 43 %). IR (range 1700–1100 cm^−1^): 1600f, 1536m, 1502d, 1474d, 1412f, 1355d, 1299f, 1247m, 1229m, 1182m, 1130f. Slow evaporation of a toluene solution yielded well‐shaped, single crystals, suitable for X‐ray analysis. Crystals collapse loosing solvent when dried in vacuo.

7c) PyterpyNO (0.08 g; 0.24 mmol) Eu(tta)_3_(dme) (0.22 g; 0.24 mmol) and Y(tta)_3_(dme) (0.20 g; 0.24 mmol) were dissolved in toluene (40 mL). The light yellow solution, concentrated to a small volume (10 mL) was heated at 80 °C to dissolve the precipitate and slowly cooled to room temperature, yielding well‐shaped, single crystals, suitable for X‐ray analysis. Crystals collapse loosing solvent when dried in vacuo (0.25 g; 54.3 % yield). IR (range 1700–1100 cm^−1^): 1600f, 1536m, 1502d, 1474d, 1412f, 1355d, 1299f, 1247m, 1229m, 1182m, 1130f.

**X‐ray Diffraction studies**: Crystals of **4**, **5**, **6**, **7 a**, **7 b** and **7 c** were selected at room temperature (293 K), sealed in glass capillaries and analysed with a Bruker Smart Breeze CCD diffractometer equipped with Mo *Kα* radiation. The lattice parameters and some collection details are summarized in Table 2. Crystals of all compounds presented the same metric of the elementary cell. After correction for Lorentz and polarization effects and for absorption, structures were solved with the ShelXT^[95]^ program by intrinsic phasing and refined with the ShelXL^[96]^ package using least squares minimisation.^[97]^ All the structures resulted isotypic and presented disorder in some CF_3_ groups, and also in the orientation of some tiophene groups, which were refined as distributed in two limit positions distinguished by different rotations about the C−C bond. Six solvent molecules (toluene) for each tetranuclear complex are present in the crystal structure of all of them. At the end of refinement of the structure of **7 a**, **7 b**, **7 c**, the difference Fourier map showed a positive electron density peak near to the position of Y atom and a negative electron density peak near to the position of europium atom. This discrepancy was attributed to some positional exchange of the two metals in the two different positions. The difference Fourier maps of compounds **4**, **5** and **6** did not show so high residual electron density peaks, and no level *a* alert was detected by checkcif examination of their final data files. However, all the six structures were furtherly refined introducing a mixed occupancy in the metal positions and fixing to one the total occupancy for each position. The final reliability factors for all the reported structures are listed in Table 2.

**Table 2:** Crystal data and refinement summaries for **4**, **5**, **6** and **7**.

Identification code	**4⋅**6 toluene	**5⋅**6 toluene	**6⋅**6 toluene	**7 a⋅**6 toluene	**7 b⋅**6 toluene	**7 c⋅**6 toluene
CCDC number	2159058.	2159063	2159061	2159059.	2159062	2159060
Empirical formula	C_178_H_124_F_36_La_2_N_8_O_26_S_12_Y_2_	C_178_H_124_Dy_2_F_36_La_2_N_8_O_26_S_12_	C_178_H_124_Eu_2_F_36_La_2_N_8_O_26_S_12_	C_178_H_124_Eu_2_F_36_N_8_O_26_S_12_Y_2_	C_178_H_124_Eu_2_F_36_N_8_O_26_S_12_Y_2_	C_178_H_124_Eu_2_F_36_N_8_O_26_S_12_Y_2_
Formula weight	4315.20	4462.38	4441.30	4341.30	4341.30	4341.30
Crystal system	Monoclinic	Monoclinic	Monoclinic	Monoclinic	Monoclinic	Monoclinic
Space group	*P* 2_1_/*n*	*P* 2_1_/*n*	*P* 2_1_/*n*	*P* 2_1_/*n*	*P* 2_1_/*n*	*P* 2_1_/*n*
*a* (Å)	13.7727(4)	13.7990(4)	13.8381(6)	13.8001(9)	13.7868(7)	13.7875(5)
*b* (Å)	38.9733(13)	38.9844(12)	39.0648(16)	38.742(3)	38.7635(17)	38.8068(13)
*c* (Å)	18.5708(6)	18.5605(6)	18.6412(8)	18.5480(14)	18.5467(9)	18.5383(6)
*β* (°)	103.4820(10)	103.4980(10)	103.602(2)	103.689(3)	103.647(2)	103.6960(10)
Volume (Å^3^)	9693.5(5)	9708.7(5)	9794.5(7)	9634.8(12)	9632.0(8)	9636.9(6)
*Z*	2	2	2	2	2	2
*ρ*_calc_ (g cm^−1^)	1.478	1.526	1.506	1.496	1.497	1.496
*μ* (mm^−1^)	1.254	1.421	1.286	1.469	1.469	1.469
*F*(000)	4328	4436	4424	4352	4352	4352
*θ* range (°)	2.38 to 26.5	1.05 to 26.4	1.04 to 26.4	2.39 to 26.0	2.19 to 26.1	2.35 to 26.5
Reflections collected	84913	91572	90468	136562	138568	88328
Independent reflections	19842	19788	19986	18379	18965	19730
Goodness‐of‐fit on *F* ^2^	1.007	1.030	1.023	1.038	1.038	1.029
Final *R*_1_ [*I*≥2*σ*(*I*)]	0.0636	0.0454	0.0464	0.0589	0.0621	0.0563
Final *wR*_2_ [*I*≥2*σ*(*I*)]	0.1936	0.1324	0.1374	0.1513	0.1582	0.1525
Final *R*_1_ [all data]	0.0969	0.0614	0.0634	0.0893	0.1171	0.1026
Final *wR*_2_ [all data]	0.2196	0.1439	0.1502	0.1677	0.1856	0.1762
Largest peak/hole (*e* Å^−3^)	1.039, −0.848	0.694, −0.579	0.882, −0.554	0.666, −0.566	0.695, −0.918	0.644, −0.498

Other control calculations were performed with the programs contained in the suite WINGX.^[98]^

Deposition Numbers 2159058 (for **4**), 2159059 (for **7 a**), 2159060 (for **7 c**), 2159061 (for **6**), 2159062 (for **7 b**) 2159063 (for **5**) contain the supplementary crystallographic data for this paper. These data are provided free of charge by the joint Cambridge Crystallographic Data Centre and Fachinformationszentrum Karlsruhe Access Structures service

## Conflict of interest

The authors declare no conflict of interest.

7

## Supporting information

As a service to our authors and readers, this journal provides supporting information supplied by the authors. Such materials are peer reviewed and may be re‐organized for online delivery, but are not copy‐edited or typeset. Technical support issues arising from supporting information (other than missing files) should be addressed to the authors.

Supporting Information

## Data Availability

The data that support the findings of this study are available in the supplementary material of this article.

## Footnotes

n1 It must be stressed that, due to the absence of any observable magnetic interaction between the Dy^3+^ ions in positions 1 and 2, the present approach is not able to address additional species, arising from compositional disorder, like Dy_4_, La_4_ or Dy_3_La, the lanthanum(III) ion sitting in the external position 2. The presence of these species seems however unlikely, when comparing the results arising from the present magnetic analysis with the ones taken from X‐ray diffractometry data (see below).
