# Supplementary material for: Size Selectivity in Heterolanthanide Molecular Complexes with a Ditopic Ligand
Source: Chemistry. 2022 Nov 24;29(3):e202202823. doi: 10.1002/chem.202202823 (PMC10100000; doi:10.1002/chem.202202823)
Supplement: Supplementary file 1 — Supporting Information [file CHEM-29-0-s001.pdf]

Chemistry–A European Journal

Supporting Information

Size Selectivity in Heterolanthanide Molecular Complexes with a Ditopic Ligand

Luca Bellucci, Lorenzo Fioravanti, Lidia Armelao, Gregorio Bottaro,* Fabio Marchetti, Francesco Pineider, Giordano Poneti,* Simona Samaritani, and Luca Labella*

Electronic Supplementary Information

Chemistry A European Journal

Table of contents

Characterization Data (List of Figures and Tables):

Figure S1. Comparison between the ATR-IR spectrum of compounds **4** with that of the tetranuclear yttrium homometallic complex.

Figure S2. ATR-IR spectra of compounds **4-7**

Figure S3. Comparison between ^{19}F-NMR spectra of **6** and of the homometallic compounds of europium and lanthanum.[1]

Figure S4. ^{19}F-NMR spectra for progressively increased $Eu(tta)_3(dme)$ / pyterpyNO molar ratios (Eu: pyterpyNO). The dashed grey line represents the initial position of $[Eu(tta)_3]_2(pyterpyNO)_2$ ^{19}F signal (dinuclear complex). The table on the right reports the integrated areas of the two signals for different Eu / pyterpyNO molar ratio.

Figure S5. Comparison between ^{19}F-NMR spectra of **7** and of the homometallic compounds of yttrium and europium.

Figure S6. ^{89}Y-NMR spectrum of **4**.

Figure S7. Isothermal magnetization curves, measured at 2.0 (blue circles) and 4.0 K (red circles). Lines are guide to the eye.

Figure S8. Graphical comparison of the first coordination spheres of the of the lanthanides(III) ions in position 1 in complexes Dy_2 and Y_4.[1]

Figure S9. Comparison of the temperature dependence of the dynamic magnetic susceptibility of **5**, measured for 11 logarithmically spaced frequencies of the oscillating field, without (left) and with a 1 kOe static field applied (right). Lines reported in the frequency dependent plots (lower) are best fits using the extended Debye model, as commented in the main text.

Figure S10. Temperature dependence of the magnetic relaxation times of compound **5**, measured with no static field applied (empty dots) and 1 kOe static field (full dots). The best fitting functions arising from the model described in the text are reported as black lines.

Figure S11. Isothermal frequency dependence of the molar out-of-phase (χ''_M) magnetic susceptibility of compound **5**, measured with different static fields applied at 5.0 K. The lines are guide to the eye.

Figure S12. Comparison of the temperature dependence of the out-of-phase dynamic magnetic susceptibility of **5** (full dots, below) and Dy_4 (empty dots, above), measured for 11 logarithmically spaced frequencies of the oscillating field, without (*left*) and with a 1 kOe static field applied (*right*). Lines are guides to the eye.

Figure S13. Photoluminescence spectra of millimolar solutions of $Eu_2(tta)_6(pyterpyNO)_2$ and $Eu_4(tta)_{12}(pyterpyNO)_2$ homometallic complexes in toluene. The spectra were collected exciting the samples at 370 nm (maximum of the excitation spectra) and their integrated areas were normalized.

Table S1. Structural parameters of the first coordination sphere of Y_2 and Dy_4.[1]

References

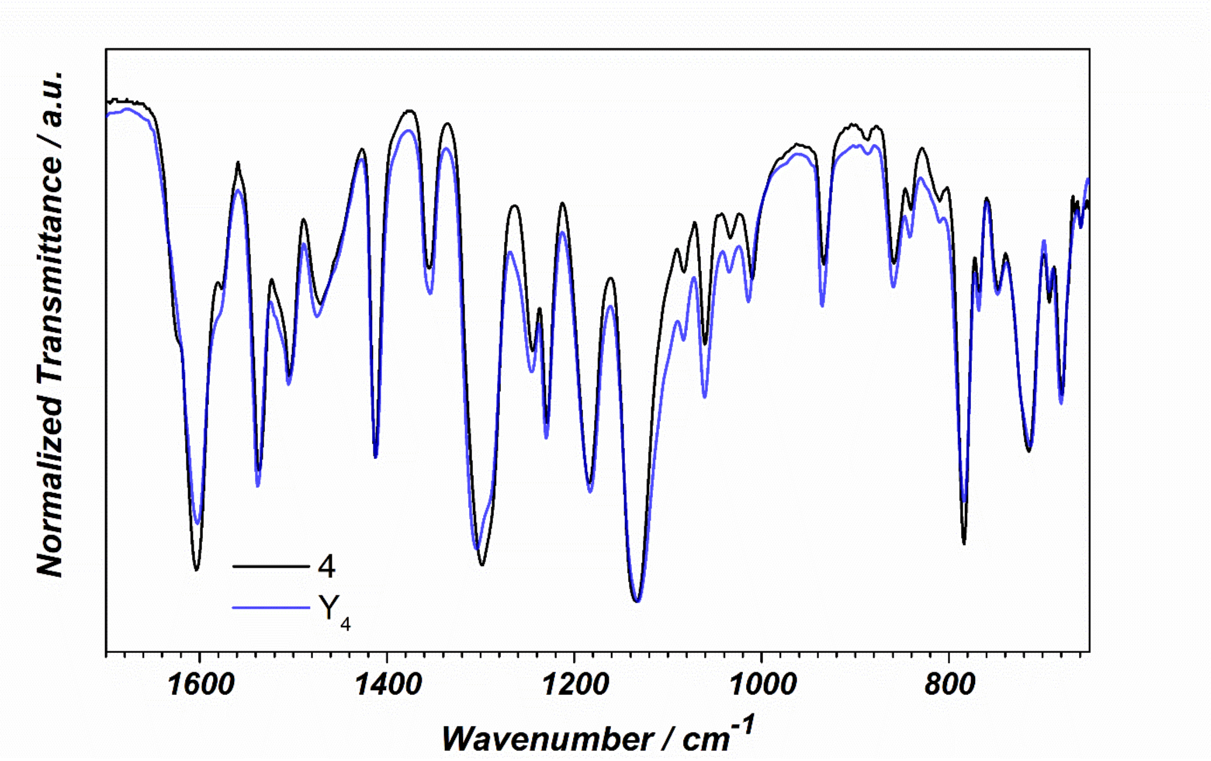

Figure S1. Comparison between the ATR-IR spectrum of compounds **4** with that of the tetranuclear yttrium homometallic complex.

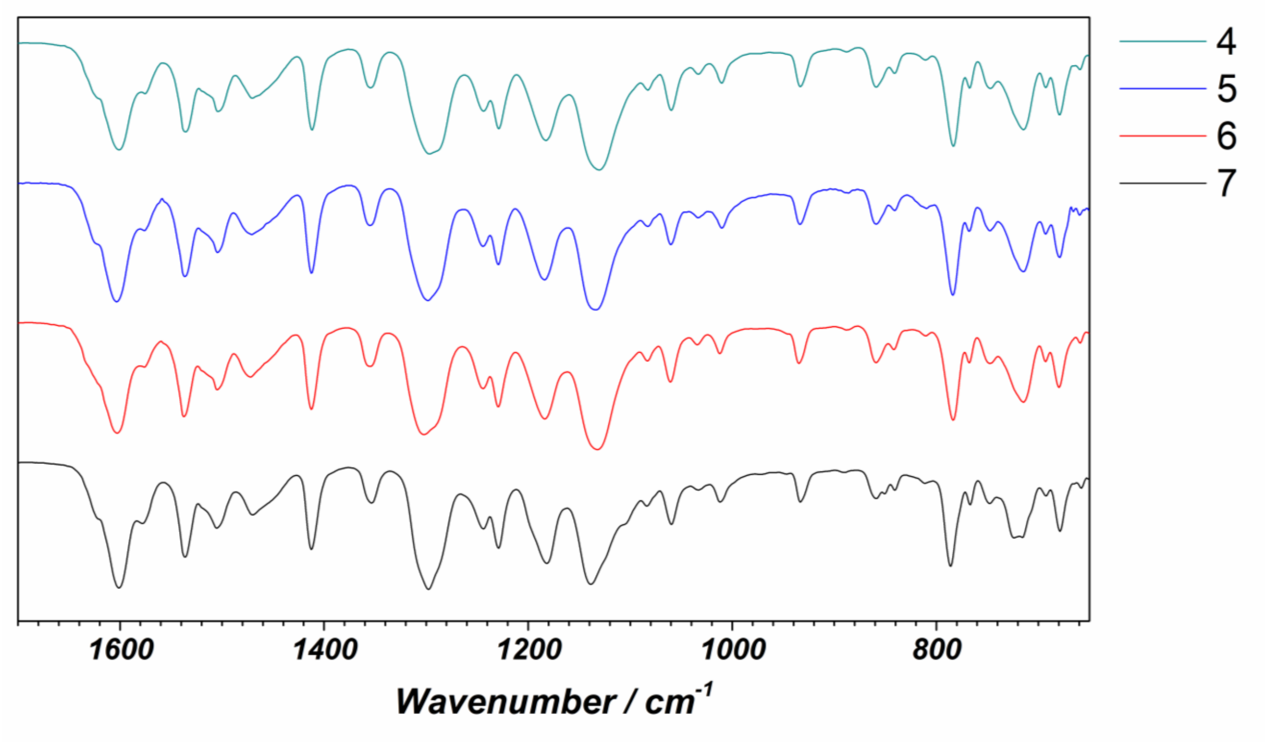

Figure S2. ATR-IR spectra of compounds **4-7**

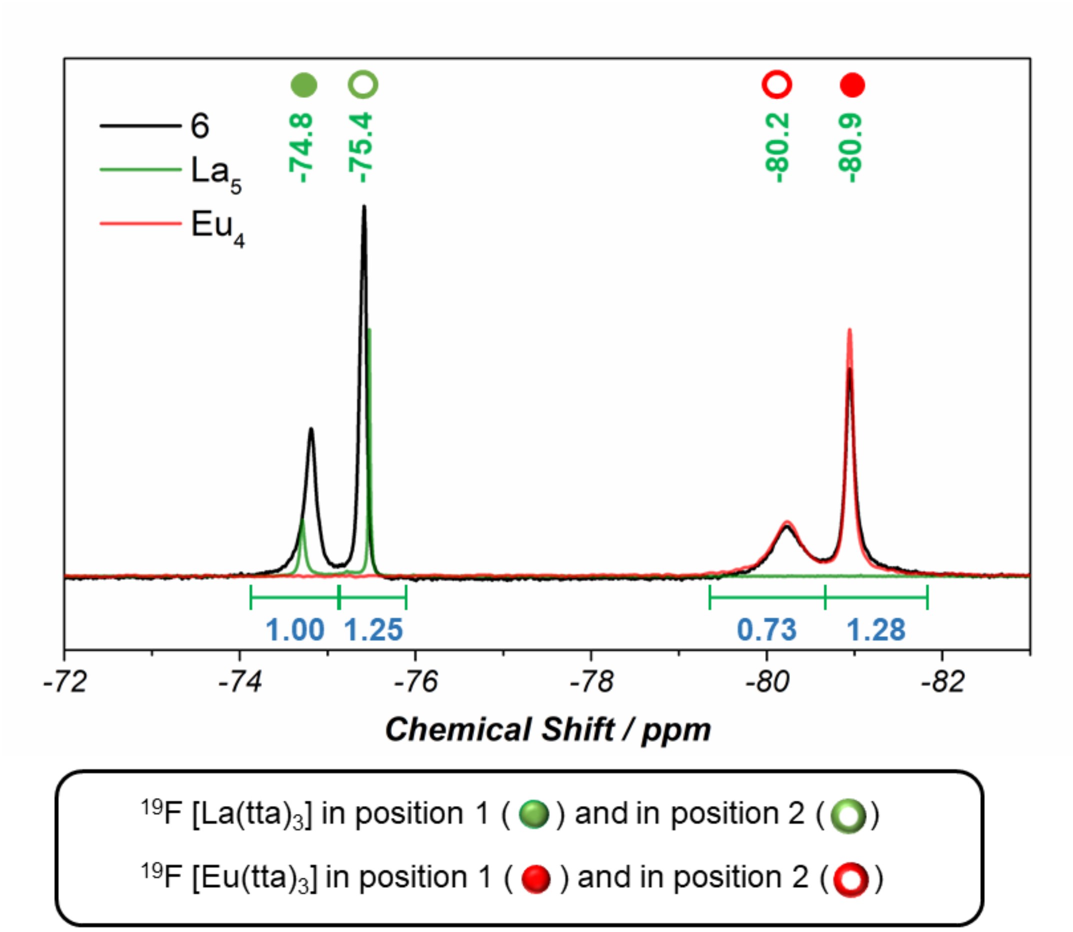

Figure S3. Comparison between ^{19}F-NMR spectra of **6** and of the homometallic compounds of europium and lanthanum.

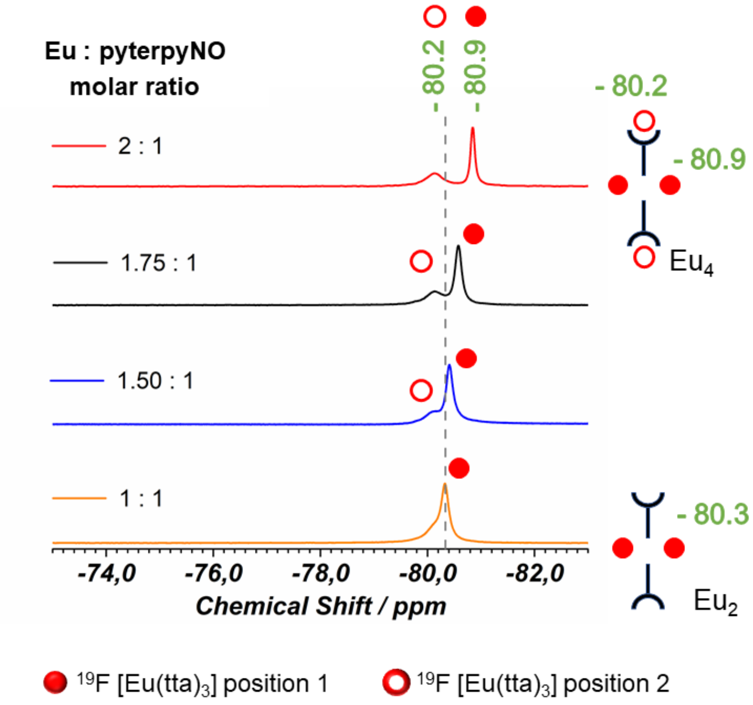

Eu : pyterpyNO molar ratio	Area ^{19}F position 1	Area ^{19}F position 2
1 : 1	0.03	1.00
1.5 : 1	0.30	1.00
1.75 : 1	0.60	1.00
2 : 1	0.90	1.00

Figure S4. ^{19}F-NMR spectra for progressively increased $Eu(tta)_3(dme)$ / pyterpyNO molar ratios (Eu: pyterpyNO). The dashed grey line represents the initial position of $[Eu(tta)_3]_2(pyterpyNO)_2$ ^{19}F signal (dinuclear complex). The table on the right reports the integrated areas of the two signals for different Eu / pyterpyNO molar ratio

In **Figure S4**, signals at -80.2 and -80.9 ppm have been attributed to *N*- and *O*-coordinated europium respectively although in the dinuclear europium complex the single signal was at -80.3 ppm. Attribution can be rationalized observing that gradually adding europium centres to the europium dinuclear complex a consistent displacement of that signal occurred towards the position at -80.9 ppm present in the tetranuclear compound.

The comparison between the integrated areas of the two signals during the addition of europium centres confirms the attribution of the two signals.

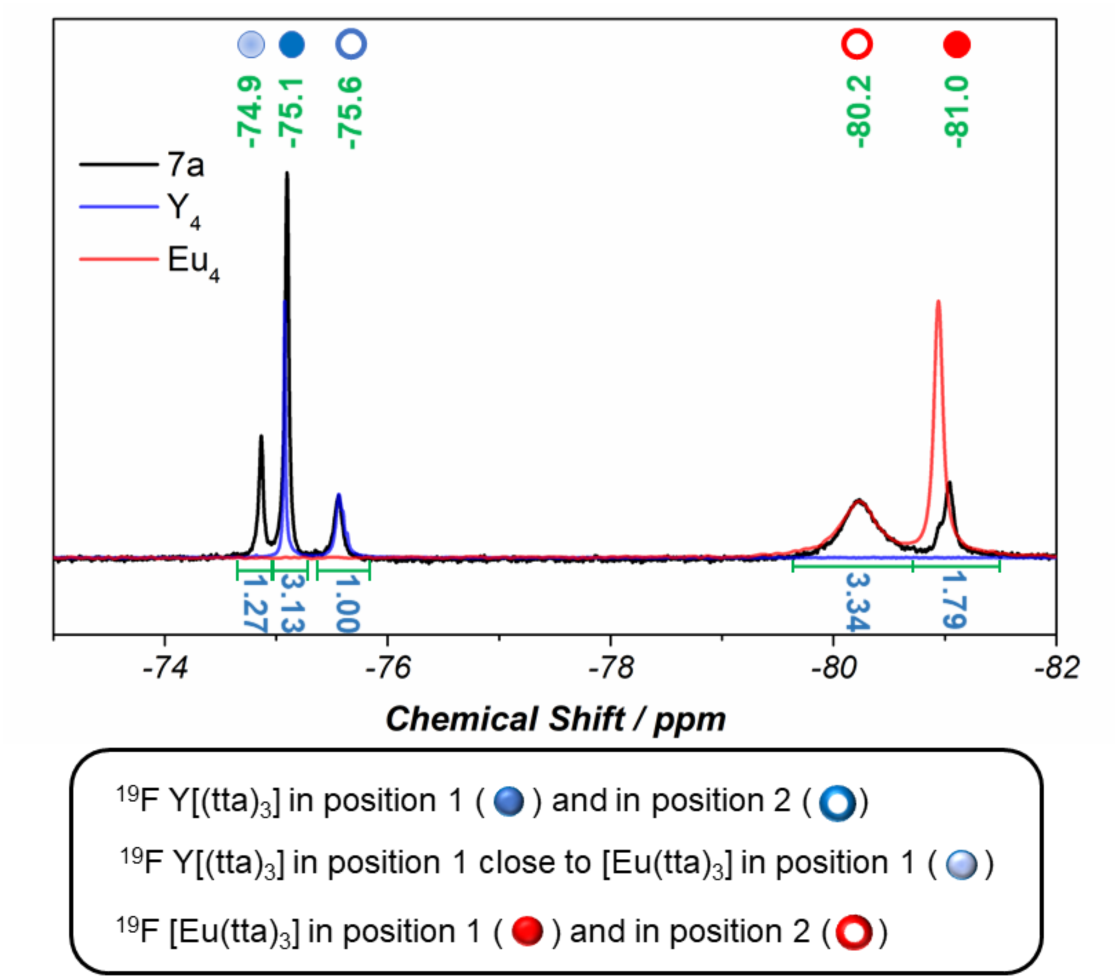

Figure S5. Comparison between ^{19}F-NMR spectra of **7** and of the homometallic compounds of yttrium and europium.

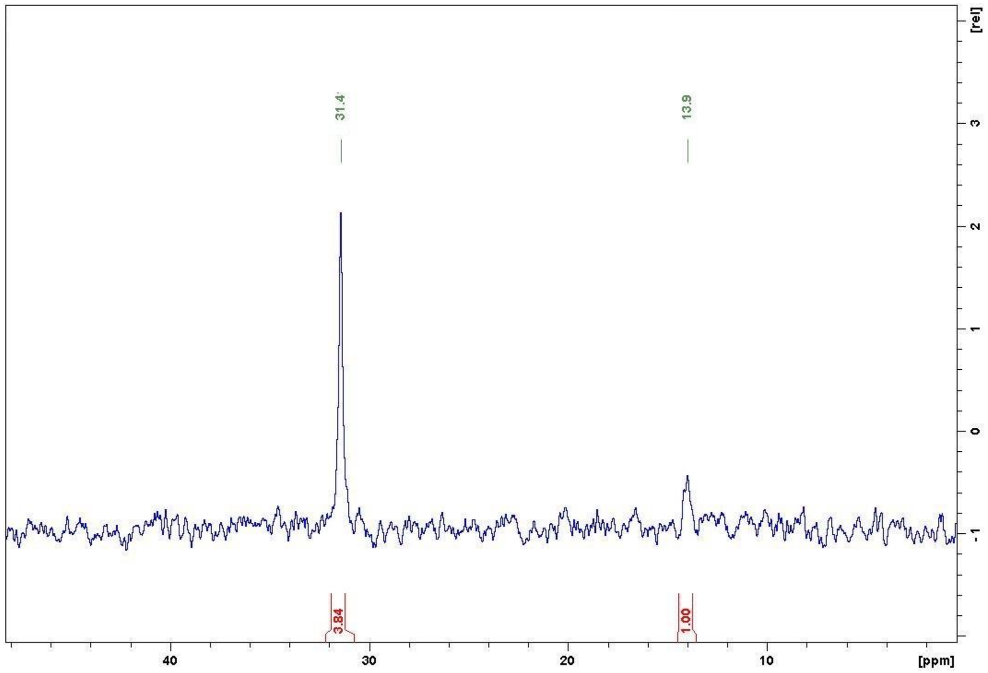

Figure S6. ^{89}Y-NMR spectrum of **4**.

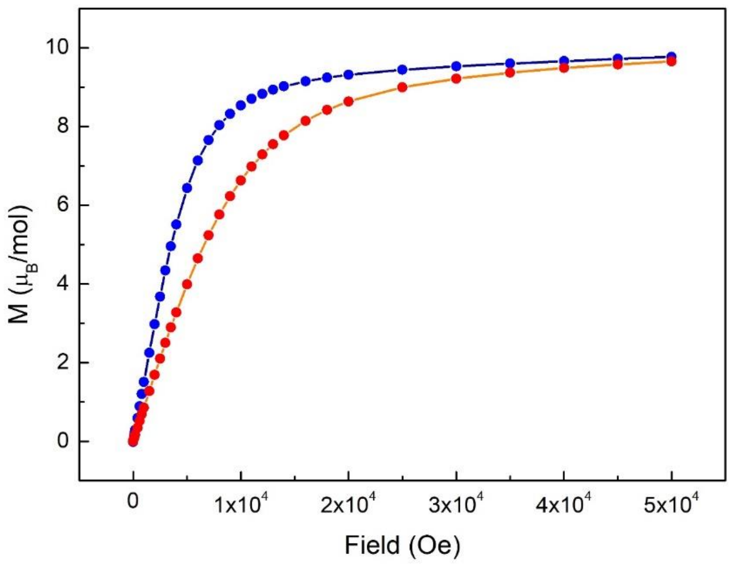

Figure S7. Isothermal magnetization curves, measured at 2.0 (blue circles) and 4.0 K (red circles). Lines are guide to the eye.

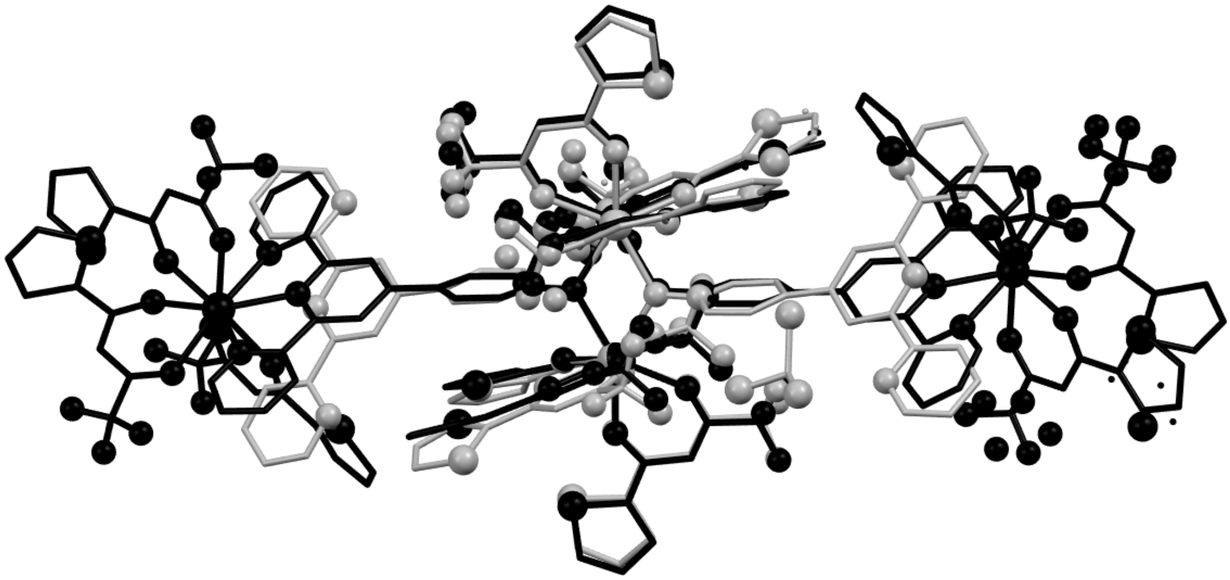

Figure S8. Graphical comparison of the first coordination spheres of the lanthanides(III) ions in position 1 in complexes Dy_2 and Y_4.[1]

Table S1. Structural parameters of the first coordination sphere of Y_2 and Dy_4.[1]

Y_2		Dy_4	
**Bond lengths (Å)**			
Y1–O1	2.332(3)	Dy1–O1	2.315(4)
Y1–O2	2.330(2)	Dy1–O2	2.322(4)
Y1–O3	2.295(3)	Dy1–O3	2.335(3)
Y1–O4	2.335(3)	Dy1–O4	2.355(3)
Y1–O5	2.348(2)	Dy1–O5	2.343(3)
Y1–O6	2.303(2)	Dy1–O6	2.306(3)
Y1–O7	2.400(2)	Dy1–O7	2.427(3)
**Bond angles (°)**			
Y1-O7-Y1	117.51(8)	Dy1-O7-Dy1A	118.65(10)
O3-Y1-O1	78.13(9)	O1-Dy1-O2	73.0(1)
O3-Y1-O2	100.95(9)	O1- Dy1-O3	77.2(1)
O3-Y1-O4	72.65(0)	O1- Dy1-O4	108.0(1)
O3-Y1-O5	73.45(9)	O1- Dy1-O5	74.8(1)
O3-Y1-O6	90.1(9)	O1- Dy1-O6	86.7(1)
O3-Y1-O7	148.44(9)	O1- Dy1-O7	147.7(1)
**Torsion angles (°)**			
O3-Y1-O7-Y1*	166.1(1)	O1-Dy1-O7-Dy1#	164.9(2)

symmetry operation to generate equivalent atom: * = 1-x, 1-y, 1-z. # = 2-x, 1-y, 1-z.

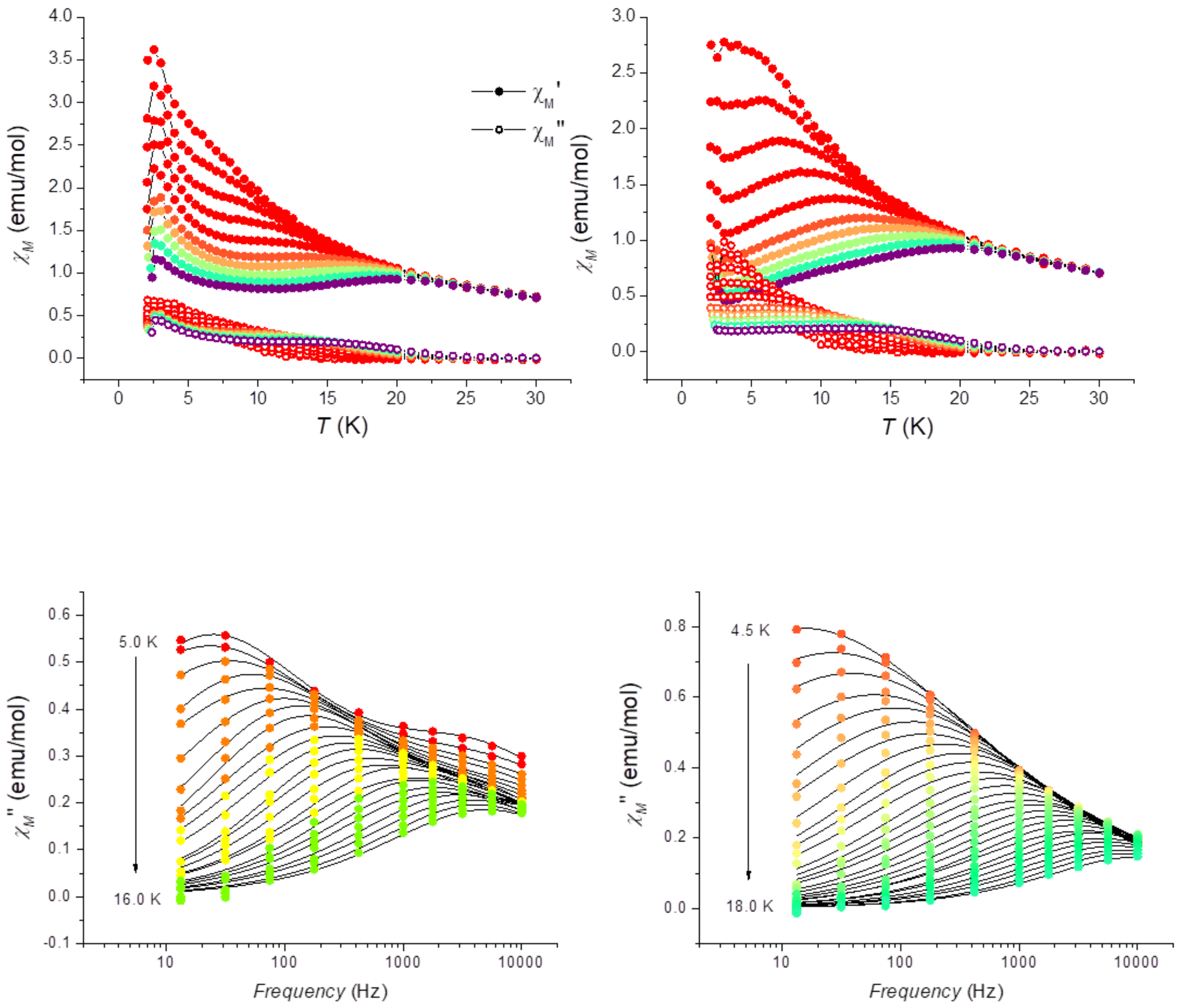

Figure S9. Comparison of the temperature dependence of the dynamic magnetic susceptibility of **5**, measured for 11 logarithmically spaced frequencies of the oscillating field, without (*left*) and with a 1 kOe static field applied (*right*). Lines reported in the frequency dependent plots (lower) are best fits using the extended Debye model, as commented in the main text.

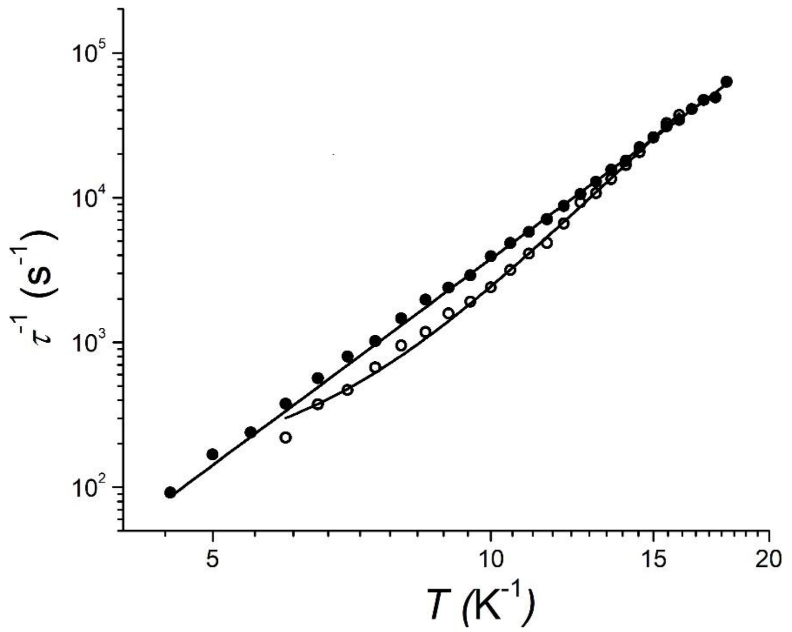

Figure S10. Temperature dependence of the magnetic relaxation times of compound **5**, measured with no static field applied (empty dots) and 1 kOe static field (full dots). The best fitting functions arising from the model described in the text are reported as black lines.

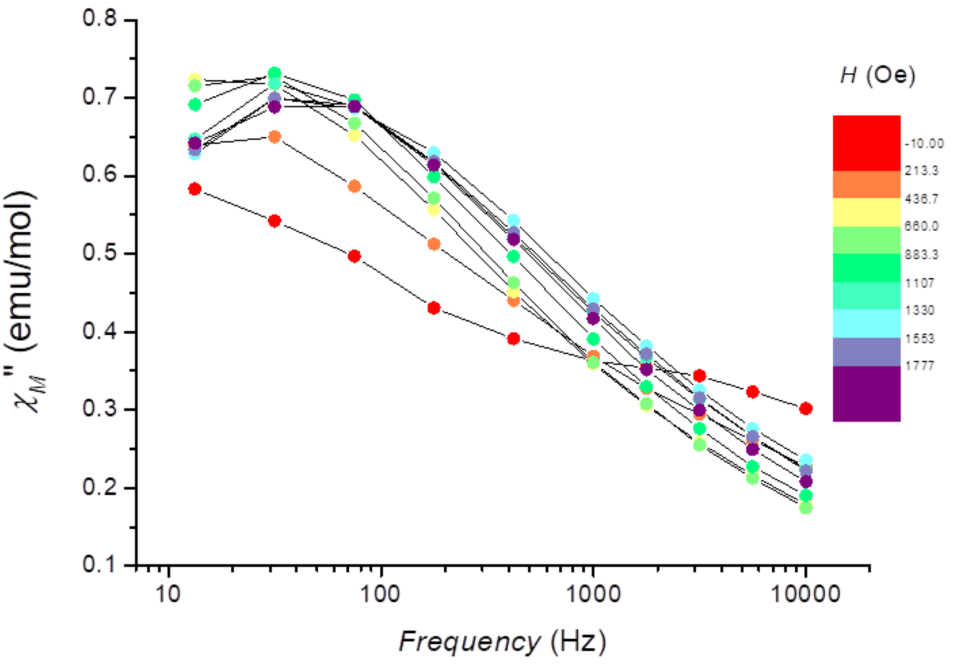

Figure S11. Isothermal frequency dependence of the molar out-of-phase (χ''_M) magnetic susceptibility of compound **5**, measured with different static fields applied at 5.0 K. The lines are guide to the eye.

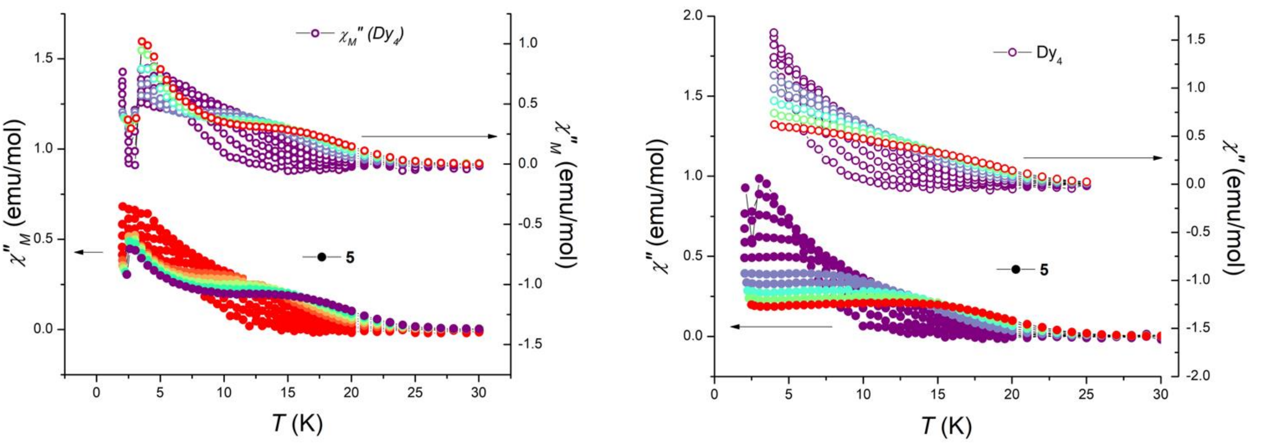

Figure S12. Comparison of the temperature dependence of the out-of-phase dynamic magnetic susceptibility of **5** (full dots, below) and Dy_4 (empty dots, above), measured for 11 logarithmically spaced frequencies of the oscillating field, without (*left*) and with a 1 kOe static field applied (*right*). Lines are guides to the eye.

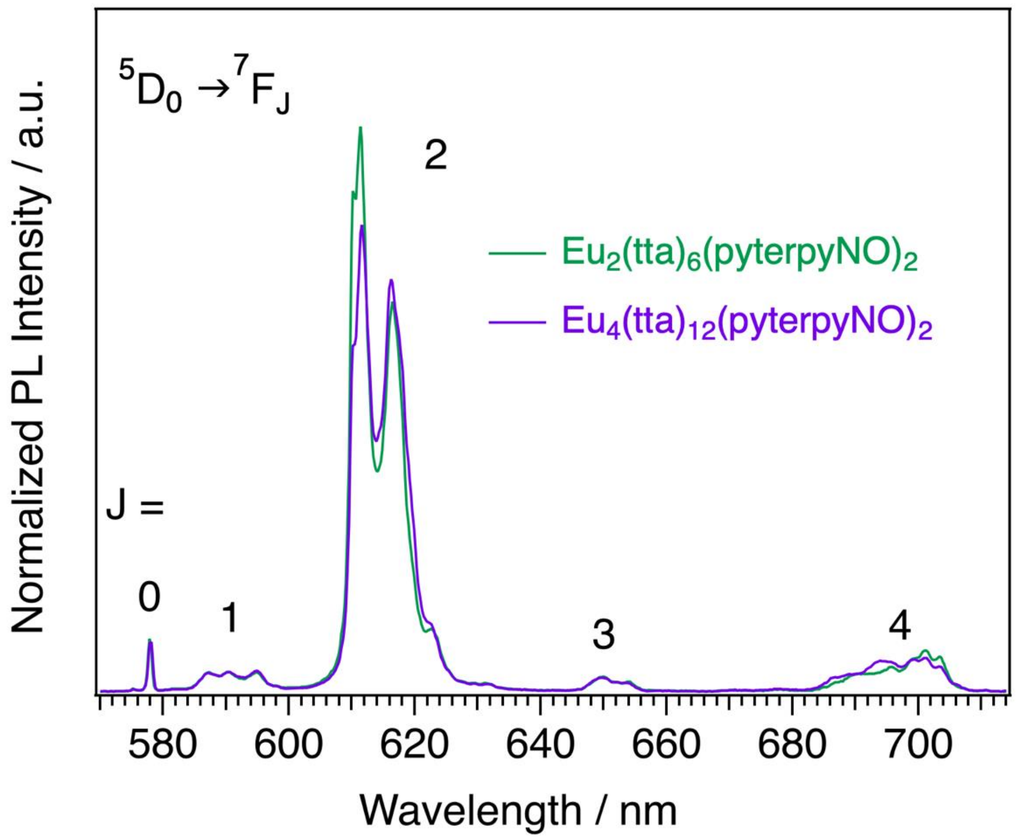

Figure S13. Photoluminescence spectra of millimolar solutions of $Eu_2(tta)_6(pyterpyNO)_2$ and $Eu_4(tta)_{12}(pyterpyNO)_2$ homometallic complexes in toluene. The spectra were collected exciting the samples at 370 nm (maximum of the excitation spectra) and their integrated areas were normalized.

References

[1] L. Fioravanti, L. Bellucci, L. Armelao, G. Bottaro, F. Marchetti, F. Pineider, G. Poneti, S. Samaritani, L. Labella, *Inorg. Chem.* **2022**, *61*, 265-278.
